# Antisense oligonucleotide silencing of FUS expression as a therapeutic approach in amyotrophic lateral sclerosis

**DOI:** 10.1038/s41591-021-01615-z

**Published:** 2022-01-24

**Authors:** Vlad A. Korben, Alexander K. Lyashchenko, Beatriz Blanco-Redondo, Paymaan Jafar-Nejad, Neil A. Shneider

**Affiliations:** 1 Department of Neurology, Center for Motor Neuron Biology and Disease, Columbia University, New York, NY USA; 2 Department of Pathology and Cell Biology, Columbia University, New York, NY USA; 3 Ionis Pharmaceuticals, Carlsbad, CA USA; 4 Department of Neurology, Eleanor and Lou Gehrig ALS Center, Columbia University, New York, NY USA; 5 Present Address: Rudolf-Schönheimer Institute for Biochemistry, Leipzig University, Leipzig, Germany

**Keywords:** Amyotrophic lateral sclerosis, Neurodegeneration

## Abstract

Fused in sarcoma (FUS) is an RNA-binding protein that is genetically and pathologically associated with rare and aggressive forms of amyotrophic lateral sclerosis (ALS) and frontotemporal dementia (FTD). To explore the mechanisms by which mutant FUS causes neurodegeneration in ALS-FTD, we generated a series of FUS knock-in mouse lines that express the equivalent of ALS-associated mutant FUSP525L and FUSΔEX14 protein. In FUS mutant mice, we show progressive, age-dependent motor neuron loss as a consequence of a dose-dependent gain of toxic function, associated with the insolubility of FUS and related RNA-binding proteins. In this disease-relevant mouse model of ALS-FUS, we show that ION363, a non-allele-specific FUS antisense oligonucleotide, efficiently silences Fus and reduces postnatal levels of FUS protein in the brain and spinal cord, delaying motor neuron degeneration. In a patient with ALS with a FUSP525L mutation, we provide preliminary evidence that repeated intrathecal infusions of ION363 lower wild-type and mutant FUS levels in the central nervous system, resulting in a marked reduction in the burden of FUS aggregates that are a pathological hallmark of disease. In mouse genetic and human clinical studies, we provide evidence in support of FUS silencing as a therapeutic strategy in FUS-dependent ALS and FTD.

## Abstract (web summary)

An antisense oligonucleotide targeting the RNA-binding protein FUS transcript lowers FUS levels in mice and in the central nervous system of a single patient with FUS-dependent ALS

## Main

ALS is a fatal neurological disorder characterized primarily by the degeneration of corticospinal, bulbar and spinal motor neurons (MNs), leading to paralysis and death^1^. In most cases, the cause of ALS is unknown; however, pathogenic mutations have been identified in more than 25 genes and account for approximately 15% of cases^2–4^. The genetic complexity of ALS underlies wide phenotypic variability in the MN phenotype in terms of the age and site of disease onset, the balance of upper and lower MN findings and the rate of disease progression.

Mutations in *FUS* are associated with the most aggressive, early-onset forms of ALS^5,6^ as well as rare forms of FTD^7,8^. Like TDP-43, MATR3 and hnRNP A1, to which it is structurally and functionally related, FUS is one of several RNA-binding proteins (RBPs) that have been implicated in ALS. It is a ubiquitously expressed, predominantly nuclear protein that functions in DNA repair and several aspects of RNA metabolism, including transcription, pre-mRNA splicing, mRNA transport, stability and translation as well as the processing of microRNAs and other non-coding RNAs^9^. To date, more than 50 different FUS mutations have been identified in patients with ALS^10^, which together account for approximately 4% of familial cases and fewer than 2% of patients with sporadic ALS^10,11^. The functional consequences of these ALS-associated mutations on FUS are not known; however, although FUS loss of function is not sufficient to cause MN degeneration in vivo^12^, a deficiency in FUS activity might contribute to the pathogenesis of ALS. Strong evidence supports a toxic gain-of-function mechanism in ALS-FUS^13^ that is related to the biophysical properties of FUS and related, ALS-associated RBPs that lead to liquid–liquid phase separation (LLPS) and the consequent formation of abnormal assemblies that underlie neurodegeneration in FUS-dependent ALS and related forms of FTD^14^.

To develop a faithful model of ALS-FUS to explore disease mechanisms, identify therapeutic targets and test therapeutic candidates, we generated a series of FUS knock-in mouse lines in which ALS-causing mutations were targeted directly to the endogenous mouse *Fus* locus. We provide genetic evidence that, despite a partial loss of function associated with the equivalents of the human *FUS*^P525L^ mutation (mFus^P517L^) and a truncation mutation (G466VfsX14 or Δ14) associated with rapidly progressive, juvenile-onset ALS^15^, expression of these mutant forms of FUS in vivo at physiological levels leads to progressive, age-dependent MN degeneration that is dose dependent and selective for MN subpopulations known to be preferentially vulnerable in patients with ALS and related mouse models of familial ALS. Using a conditional allele of Δ14, we show that FUS-dependent MN degeneration is a cell-autonomous process, driving secondary inflammatory changes that do not depend on mutant FUS expression in the reactive cells but might contribute to neurodegeneration. In these knock-in mouse models of ALS-FUS, toxicity correlates with the degree of insolubility of FUS and other RBPs, which is associated with functional deficiency of these related, phase-separating proteins.

Finally, we show that an experimental ASO that targets the FUS transcript (ION363) effectively silences wild-type and mutant FUS in the brain and spinal cord of P517L and Δ14 heterozygous mice. Consistent with our model of a dose-dependent, gain-of-function mechanism of disease, we show that a single intracerebroventricular (ICV) injection of this FUS ASO delays the onset of MN degeneration in a conditional compound heterozygous mutant FUS mouse with an accelerated, ALS-like phenotype. In a first-in-human study, we found that repeated intrathecal (IT) administration of ION363 in a patient with ALS-FUS with a FUS^P525L^ mutation results in the marked suppression of FUS expression in the brain and spinal cord and the reduction of the FUS aggregates that are the pathological hallmark of this disease. We provide evidence to support the clinical application of ION363 in the treatment of ALS-FUS and related FUS-dependent proteinopathies.

## Results

### Selective MN degeneration in knock-in mice modeling ALS-FUS

**Fig. 1:**
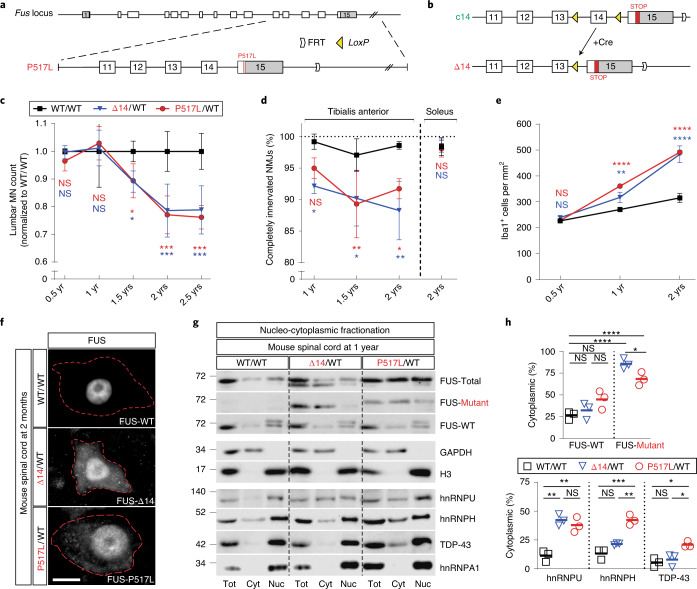
Selective MN degeneration and mislocalization of FUS and other RBPs to the cytoplasm in knock-in mice expressing ALS-associated mutant FUS. **a**, Schematic of the creation of mutant *Fus* knock-in alleles. Top: the murine *Fus* locus. Bottom: the P517L targeting vector used for homologous recombination. Exons are represented as gray (5′ UTR and 3′ UTR) and white (coding sequence) rectangles. FRT site downstream of the 3′ UTR is the ‘scar’ left after removal of NEO resistance cassette. **b**, Conditional c14 allele (top) has exon 14 flanked by *LoxP* sites (yellow triangles). Cre recombinase-dependent recombination at the *LoxP* sites in c14 excises exon 14 and converts it to the mutant Δ14 allele. In addition, part of the mouse exon 15 (red rectangle) is ‘humanized’—replaced with the corresponding human exon 15 sequence. This modification does not affect the in-frame protein sequence (c14 allele produces wild-type mouse FUS protein) but alters the Δ14 C-terminal amino acid sequence to mirror the out-of-frame reading of human G466VfsX14 mutant exon 15. **c**, Numbers of ChAT-positive MNs at lumbar levels 4 and 5 in WT/WT (black), P517L/WT (red) and Δ14/WT (blue) animals normalized to the wild-type controls. *n* = 3 animals per group at 1 and 2 years and *n* = 5 animals per group at 1.5 years. **d**, Percentage of completely innervated NMJs (that is, not partially or completely denervated) in tibialis anterior (left) and soleus (right) muscles of WT/WT (black), P517L/WT (red) and Δ14/WT (blue) animals. *n* = 3 animals per group at 1 and 2 years and *n* = 4 animals per group at 1.5 years. **e**, Density of Iba1-positive microglial cells at lumbar levels 4 and 5 in WT/WT (black), P517L/WT (red) and Δ14/WT (blue) animals. *n* = 3 animals per group. **f**, Representative images of MNs from spinal cord sections of 2-month-old WT/WT, Δ14/WT and P517L/WT animals stained with FUS-Abcam[1-50], FUS-Δ14 and FUS-P517L antibodies, respectively. Red dotted lines outline MN somata. Scale bar, 10 µm. **g**,**h**, Blot (**g**) and quantification (**h**) of nucleo-cytoplasmic fractionation of brain tissue of 1-year-old wild-type (WT/WT) and heterozygous mutant (P517L/WT and Δ14/WT) animals showing mislocalization of mutant FUS and other RBPs to the cytoplasm in the mutant mice. *P < 0.05, **P < 0.01 and ***P < 0.001, using one-way ANOVA with Tukey’s post hoc test. *n* = 3 for all genotypes. Individual values and means are shown. For **c**, **d** and **e**: *P < 0.05, **P < 0.01 and ***P < 0.001, using two-way ANOVA with Tukey’s post hoc test. Data are shown as mean ± s.d. NS, not significant. Source data

To study the effects of ALS-causing mutations on normal FUS function and to model the toxicity of mutant FUS in vivo, we introduced two ALS-FUS mutations associated with a rapidly progressive, juvenile-onset form of ALS into the mouse *Fus* locus (Fig. 1a,b; see Methods for details). The mouse FUS P517L mutation (Fig. 1a) is equivalent to the human FUS P525L allele^16^. The mouse FUS Δ14 mutation (Fig. 1b) is equivalent to the human G466VfsX14 C-terminal FUS truncation mutation^15^, which causes skipping of *FUS* exon 14. To enable conditional expression of the FUS Δ14 mutant, we generated the wild-type FUS-expressing c14 allele, which can be converted to the truncated mutant FUS-producing Δ14 allele via Cre-mediated recombination (Fig. 1b).

**Extended Data Fig. 1:**
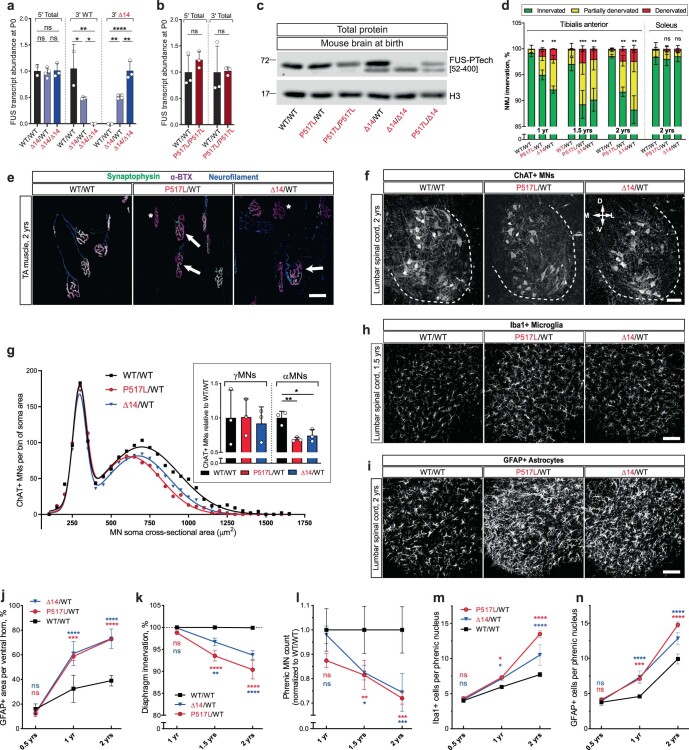
ALS-like pathology in mutant FUS knock-in mice. **(a)** RT-qPCR quantification of newborn (P0) brain *Fus* transcripts in wild type (WT/WT), *Fus* Δ14 heterozygous (Δ14/WT) and *Fus* Δ14 homozygous (Δ14/Δ14) animals using primers positioned in exons 1–3 of the *Fus* transcript that amplify WT and Δ14 equally (5’ Total), primers spanning exon 13–14 junction specific to the WT *Fus* transcript (3’ WT), or primers spanning exon 13–15 junction specific to the Δ14 *Fus* transcript (3’ Δ14). Transcript abundances were normalized to WT/WT for ‘5’ Total’ and ‘3’ WT” and to Δ14/ Δ14 for ‘3’ Δ14’. N = 2, 4, and 3 for WT/WT, Δ14/WT, and Δ14/ Δ14 respectively. *P < 0.05, **P < 0.01 and ***P < 0.001, using one-way ANOVA with Tukey’s post hoc test. Data shown as mean ± SD. **(b)** RT-qPCR quantification of newborn (P0) brain *Fus* transcripts in wild type (WT/WT) and *Fus* 517L homozygous (P517L/P517L) animals using primers positioned in exons 1–3 (5’ Total) or in exons 13–14 (3’ Total) of the *Fus* transcript that amplify WT and 517L equally. Transcript abundances were normalized to WT/WT. N = 3 animals of each genotype for each group. Statistical significance was assessed using Welch’s t-test. Data shown as mean ± SD. **(c)** Representative immunoblot of brain total protein extracts from newborn animals using FUS-PTech[52–400] antibody. **(d)** Percentages of NMJs classified as fully innervated (green), partially denervated (yellow), and fully denervated (red) in the tibialis anterior (left) and soleus (right) muscles in WT/WT, P517L/WT, and Δ14/WT animals. *P < 0.05, **P < 0.01 and ***P < 0.001 indicates significant differences in fully denervated NMJs between the corresponding genotype and WT controls using two-way ANOVA with Dunnett’s post hoc test. Data shown as mean ± SD. N = 3 animals per group at 1 and 2 years and 4 animals per group at 1.5 years. **(e)** Representative images of tibialis anterior (TA) muscle innervation in 2-year-old WT/WT (left), P517L/WT (middle), and Δ14/WT (right) animals. Muscle innervation was determined by co-localization of markers for motor axon terminals (anti-synaptophysin antibody, green) and post-synaptic NMJ acetylcholine receptors (fluorophore-conjugated α-bungarotoxin, or α-BTX, magenta). In addition, motor axons were labeled with anti-neurofilament antibody (blue). White asterisks indicate fully denervated motor endplates (prominent α-BTX with no overlapping synaptophysin, absent neurofilament) and white arrows indicate partially denervated NMJs (partial overlap of synaptophysin and α-BTX, presence of neurofilament). Scale bar=50 µm. **(f)** Representative images of lumbar level 5 (L5) spinal cord ventral horns of 2-year-old WT/WT (left), P517L/WT (middle), and Δ14/WT (right) animals stained with anti-ChAT antibody (white). Compass mark indicates dorsal (D), ventral (V), medial (M) or lateral (L) orientation. Scale bar=100 µm. **(g)** Histogram of MN soma cross-sectional areas of WT/WT (black), P517L/WT (red), and Δ14/WT (blue) animals. MNs with soma area ≥475 µm^2^ were classified as α and < 475 µm^2^ as γMNs. Inset shows the numbers of ChAT-positive γMNs (left) or αMNs (right) at lumbar levels 4 and 5 in 2-year-old WT/WT (black), P517L/WT (red), and Δ14/WT (blue) animals normalized to the wild type controls. *P < 0.05, **P < 0.01 and ***P < 0.001 using one-way ANOVA with Tukey’s post hoc test. Data shown as mean ± SD. N = 3 animals per group. **(h)** Representative images of lumbar level 5 (L5) spinal cord ventral horns of 1.5-year-old WT/WT (left), P517L/WT (middle), and Δ14/WT (right) animals stained with anti-Iba1 antibody (white). Scale bar=100 µm. **(i)** Representative images of lumbar level 5 (L5) spinal cord ventral horns of 2-year-old WT/WT (left), P517L/WT (middle), and Δ14/WT (right) animals stained with anti-GFAP antibody (white). Scale bar=100 µm. **(j)** Percentage of cross-sectional area that is GFAP-positive at lumbar levels 4 and 5 in WT/WT (black), P517L/WT (red), and Δ14/WT (blue) animals. **(k)** Percentage of completely innervated NMJs (*that is*, not partially or completely denervated) in the diaphragm muscles WT/WT (black), P517L/WT (red), and Δ14/WT (blue) animals. **(l)** Numbers of ChAT positive motor neurons in phrenic nucleus at cervical levels 3 through 5 in WT/WT (black), P517L/WT (red), and Δ14/WT (blue) animals normalized to the wild type controls. **(m)** Numbers of Iba1-positive microglial cells within phrenic nucleus at cervical levels 3 through 5 in WT/WT (black), P517L/WT (red), and Δ14/WT (blue) animals. **(n)** Numbers of GFAP-positive astroglial cells within phrenic nucleus at cervical levels 3 through 5 in WT/WT (black), P517L/WT (red), and Δ14/WT (blue) animals. For j-n: *P < 0.05, **P < 0.01 and ***P < 0.001, using two-way ANOVA with Tukey’s post hoc test. Data shown as mean ± SD. N = 3 animals of each genotype for each group. Source data

In initial experiments, we showed that, in heterozygous P517L/WT and Δ14/WT mutant FUS knock-in mice, the mutant alleles are expressed at physiological levels from endogenous *Fus* locus (Extended Data Fig. 1a–c). Having reproduced the genotype of patients with ALS-FUS, we asked whether these mutations are sufficient to cause selective MN degeneration in the relatively short lifespan of a mouse by examining the spinal cord and skeletal muscles of adult heterozygous mutants and wild-type controls. Using choline acetyl transferase (ChAT) as an MN marker, we found no MN loss in the lumbar spinal cord at 6 months or 1 year of age in either the P517L/WT or Δ14/WT mice (Extended Data Fig. 1f); however, at 1.5 years, we observed approximately 11% fewer MNs in both mutants compared to the wild-type animals (Fig. 1c). By 2 years of age, MN loss in both the P517L/WT and Δ14/WT mutants progressed to approximately 22% and remained at a similar level at 2.5 years (Fig. 1c). Consistent with other mouse ALS models^17^, MN loss was restricted to alpha-MNs such that, at 2 years of age, 31% and 26% of the alpha-MNs were lost in P517L/WT and Δ14/WT mutants, respectively, whereas gamma-MNs were completely spared (Extended Data Fig. 1g). Despite significant MN loss, these mice did not develop an overt motor phenotype.

Because denervation of fast-fatigable (FF) motor units in skeletal muscle precedes the spinal MN loss in patients with ALS and in SOD1, TDP-43 and FUS mouse models of ALS^12,18,19^, we next looked for evidence of denervation in the tibialis anterior (TA) muscle, which is innervated predominantly by FF MNs. At 1 year of age, we found that approximately 5% and 8% of the TA neuromuscular junctions (NMJs) were at least partially denervated in P517L/WT and Δ14/WT mutant mice, respectively, compared to 0.8% denervation observed in the wild-type mice (Fig. 1d and Extended Data Fig. 1d,e). By 1.5 years, TA denervation increased to 11% and 10% in P517L/WT and Δ14/WT mutants, respectively, and persisted at a similar level at 2 years of age (Fig. 1d). In contrast, the soleus muscle, innervated mostly by slow MNs that are relatively spared in patients with ALS and animal models, remained innervated in all 2-year-old animals (Fig. 1d).

Furthermore, as gliosis has been associated with ALS pathology in patients and mouse models^12,20,21^, we analyzed the expression and spatial distribution of Iba1 and GFAP to monitor the proliferation of microglia and astrocytes, respectively. Significant elevations in Iba1 and GFAP were observed in the spinal ventral horns of both heterozygous mutant animals compared to wild-type controls starting at 1 year of age (Fig. 1e and Extended Data Fig. 1h,i,j). Thus, both gliosis and NMJ denervation precede MN loss in this model by approximately 6 months and seem to be early manifestations of ALS pathology.

Our studies of heterozygous ALS mutant FUS knock-in mice show that a single copy of P517L or Δ14 mutant allele is sufficient to cause gliosis and NMJ denervation, followed by selective degeneration of the same MN subtypes that are preferentially involved in ALS-FUS. This pathology is not restricted to the lumbar spinal cord, as similar trends in denervation, MN degeneration and gliosis were observed in the diaphragm muscle and cervical spinal cord (Extended Data Fig. 1k–n). Together, these observations show that the knock-in mice closely replicate ALS-FUS pathology, providing a model in which to study disease-relevant mechanisms of neurodegeneration.

### Cytoplasmic mislocalization of mutant FUS in knock-in mice and ALS fibroblasts

**Extended Data Fig. 2:**
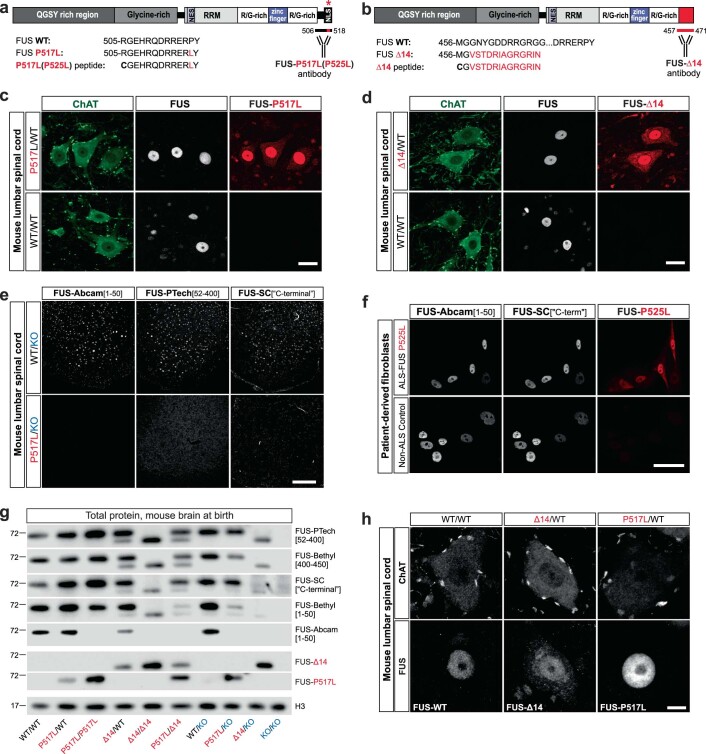
Variable reactivity of commercial antibodies with mutant FUS: characterization of mutant FUS expression and localization using novel mutant FUS-specific antibodies. (**a, b**) Schematics of P517L and Δ14 mutant FUS proteins (top). Mutations are indicated by red asterisk for P517L and red C-terminal rectangle for Δ14. Bottom shows C-terminal amino acid sequences of FUS WT protein, FUS P517L and FUS Δ14 proteins, and the P517L and Δ14 synthetic peptides used for the generation of FUS-P517L and FUS-Δ14 antibodies (bottom). Red letters in the amino acid sequences indicate the residues specific to the FUS mutants. The locations of the epitopes for FUS-P517L and FUS-Δ14 antibodies are marked by horizontal bars below the C-termini of the corresponding schematized mutant proteins (bottom right). The murine and human C-terminal FUS amino acid sequences are conserved, and the illustrated mouse sequences also correspond to the human FUS P525L and Δ14 proteins. (**c, d**) Immunostaining of adult spinal cord sections of 1-year-old heterozygous mutant (P517L/WT and Δ14/WT) and WT/WT mice using FUS-Abcam[1–50] (FUS, white) and FUS-P517L or FUS-Δ14 (red) antibodies. Note the cytoplasmic staining with FUS-P517L andFUS-Δ14 antibodies in the animals that express the corresponding mutant FUS protein (upper panels) and the absence of staining of wild type FUS protein in WT/WT animals using either the P517L or the Δ14 antibodies (bottom panels). Scale bar: 20 µm. (**e**) Representative images of spinal cord sections from WT/KO and P517L/KO animals show lack of immunoreactivity of three commercial antibodies with P517L mutant FUS protein. Scale bar=100 µm. (**f**) Immunostaining of human cultured fibroblasts from an ALS-FUS^P525L^ patient (top) and a non-ALS control (bottom) using two distinct FUSWT antibodies (white) and the FUS-P517L(P525L) antibody (red). Identical confocal microscopy settings (laser power and gain) were used for imaging the top and the bottom panels. Scale bar: 50 µm. (**g**) Denaturing immunoblot of brains of newborn mice of the indicated genotypes demonstrates variable affinities of five commercially available anti-FUS antibodies for mutant relative to wild type FUS. Note, for example, absence of visible bands for either P517L or Δ14 mutant FUS protein with the FUS-Abcam[1–50] antibody. In addition, absence of visible bands for WT protein confirms the specificity of FUS-P517L and FUS-Δ14 antibodies for mutant FUS. (**h**) Representative images of MNs from spinal cord sections of 2-year-old WT/WT (left), Δ14/WT (middle), and P517L/WT (right) animals stained with FUS-Abcam[1–50], FUS-Δ14, and FUS-P517L antibodies, respectively, show lack of obvious inclusions. Scale bar=5 µm. Source data

Using a series of novel antibodies that distinguish wild-type FUS from the mutant isoforms of the protein (Extended Data Fig. 2a–g), we observed diffuse cytoplasmic mislocalization of mutant, but not wild-type, FUS in P517L/WT and Δ14/WT animals, as well as strong nuclear staining for mutant FUS (Fig. 1f). We next quantified the cytoplasmic versus nuclear distribution of wild-type and mutant FUS by subcellular fractionation and western blot analysis. Similar to the results of immunostaining experiments, we found that a minority (~26–45%) of wild-type FUS is cytoplasmic in the spinal cords of 1-year-old wild-type, P517L/WT and Δ14/WT animals (Fig. 1g,h). By contrast, most mutant FUS (~68–86%) is cytoplasmic in P517L/WT and Δ14/WT mutants (Fig. 1h). Although mutant FUS does not appear to carry wild-type FUS into the cytoplasm, we asked if any of the RBPs known to interact directly or indirectly with FUS^22,23^ were also mislocalized. Indeed, several of these RBPs (including TDP-43, hnRNP H and hnRNP U in P517L/WT and hnRNP U in Δ14/WT) are observed at a significantly higher proportion in the cytoplasm of mutant animals compared to wild-type controls (Fig. 1g,h). Taken together, these experiments show that mutant, but not wild-type, FUS is selectively mislocalized to the cytoplasm along with interacting RBPs in P517L/WT and Δ14/WT animals.

### Dose-dependent toxicity of mutant FUS associated with RBP insolubility

**Fig. 2:**
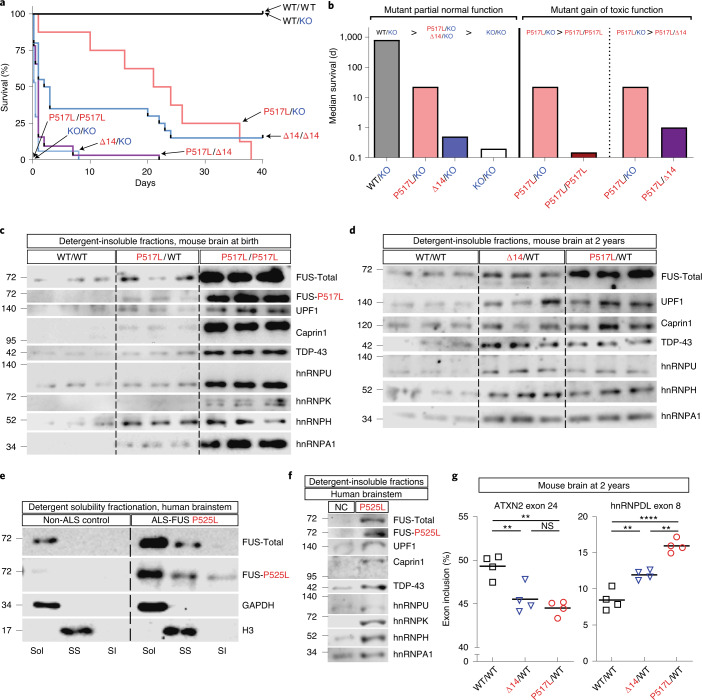
Dose-dependent toxicity and detergent insolubility of mutant FUS. **a**, Kaplan–Meier survival curves for mice with the indicated combinations of *Fus* WT, P517L, Δ14 and null-knockout alleles. **b**, Median survival of selected genotypes plotted on logarithmic scale illustrates partial functionality and dose-dependent toxicity of mutant FUS. Increased survival of P517L/KO and Δ14/KO compared with KO/KO animals shows that mutant FUS protein is able to partially rescue the null phenotype and, thus, is functional. Comparison of P517L/P517L and P517L/Δ14 versus P517L/KO animals shows that further addition of mutant FUS protein decreases survival, consistent with dose-dependent toxicity of mutant FUS protein. Median survival was estimated for P517L/P517L (0.15 d) and KO/KO (0.2 d), as most newborn pups for these genotypes were found dead and, thus, could not be accurately quantified. The bar for P517L/KO is reproduced three times for comparison purposes. **c**, Immunoblot of sarkosyl-insoluble fractions from brains of newborn *Fus* WT/WT, heterozygous P517L/WT and homozygous P517L/P517L mice. Each lane corresponds to a separate animal. **d**, Immunoblot of sarkosyl-insoluble fractions from brains of 2-year-old *Fus* WT/WT and heterozygous P517L/WT and Δ14/WT mice. Each lane corresponds to a separate animal. **e**, Immunoblot of sarkosyl solubility fractionation of human brain stems of a non-ALS control and a patient with ALS-FUS P525L. **f**, Immunoblot of sarkosyl-insoluble fractions from human brain stem samples from a non-ALS control and a patient with ALS-FUS P525L. **g**, Relative abundance of inclusion isoforms of exons regulated by hnRNPH. The observed pattern is consistent with functional deficiency of hnRNPH, which promotes inclusion of exon 24 of *ATXN2* (left) and inhibits the inclusion of exon 8 of *hnRNPDL* transcript (right). *P < 0.05, **P < 0.01 and ***P < 0.001, using one-way ANOVA with Tukey’s post hoc test. *n* = 4 animals per genotype. SI, sarkosyl insoluble; Sol, soluble (in hypotonic buffer); SS, sarkosyl soluble (in 1% sarkosyl and high salt). Source data

**Extended Data Fig. 3:**
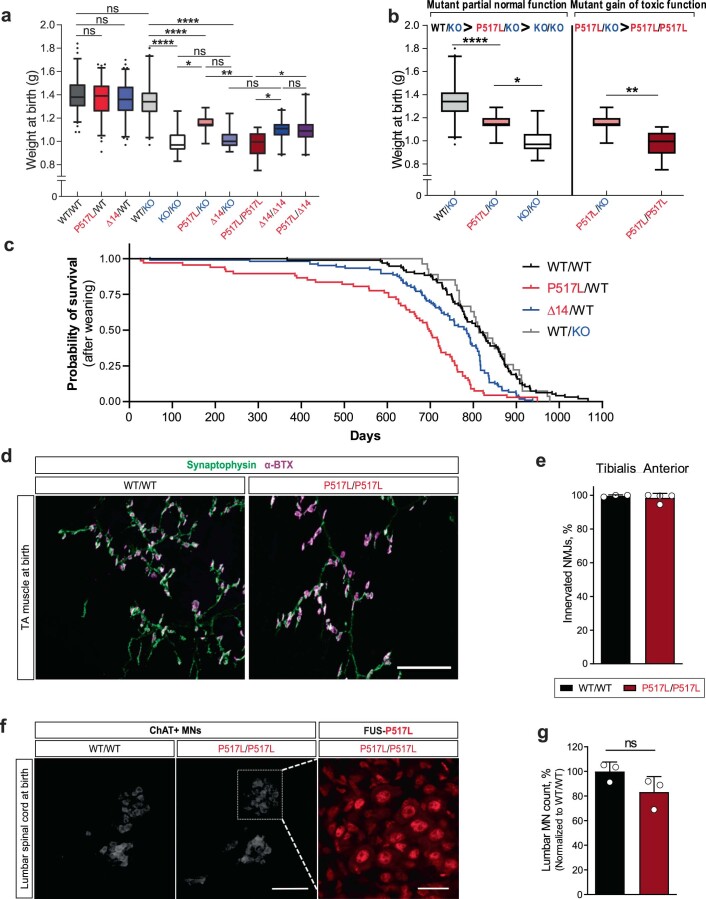
Decreased lifespan of mutant FUS heterozygous animals and absence of NMJ denervation and MN loss in newborn P517L/P517L mice. **(a)** Box (median, 25^th^ and 75^th^ percentiles) and whiskers (2.5^th^ and 97.5^th^ percentiles) plot of birth weights of mice with the indicated combinations of *Fus* WT, P517L, Δ14, and null-knockout (KO) alleles. *P < 0.05, **P < 0.01 and ***P < 0.001, using one-way ANOVA with Tukey’s post hoc test. N = 13–183 animals per genotype as indicated in Table 1. (**b**) Selected data reproduced from (a) to illustrate partial functionality and dose-dependent toxicity of mutant FUS. Increased birth weight of P517L/KO compared to KO/KO animals demonstrates that mutant FUS protein is able to partially rescue the null phenotype and thus is functional. Comparison of P517L/P517L versus P517L/KO animals demonstrates that further addition of mutant FUS protein decreases birth weight, consistent with dose-dependent toxicity of mutant FUS protein. Statistical significance was assessed in (a) as a part of a full analysis of all genotype groups. **(c**) Kaplan-Meier survival curves for successfully weaned WT/WT, P517L/WT, Δ14/WT, and WT/KO mice. Both heterozygous mutants but not WT/KO have significantly decreased median survival age compared to wild type controls. All possible pairwise comparisons were performed using Log-rank (Mantel-Cox) test and the resulting p-values were adjusted for multiple comparison using the Bonferroni correction. (**d**) NMJ staining of tibialis anterior (TA) muscle of newborn WT/WT (left) and homozygous P517L/P517L(right) animals using antibodies to pre-synaptic synaptophysin (green) and alpha-bungarotoxin (magenta, post-synaptic). Scale bar= 100 µm. **(e)** Quantification of innervated NMJs in the WT/WT (black) and homozygous P517L (dark red) animals. N = 3 for WT/WT and 4 for P517L/P517L groups respectively. **(f)** Immunofluorescence staining of lumbar level 4–5 (L4-L5) MNs in WT/WT (left) and homozygous P517L/ P517L (right) animals using anti-ChAT and anti-P517L antibodies (right inset panel). Scale bar=25 µm for the left and middle panels, 100 µm for the right panel. **(g)** Quantification of ChAT-positive neurons in lumbar levels 4–5 (L4-L5) in newborn WT/WT (black) and homozygous P517L/ P517L (dark red) animals. N = 3 animals per genotype. For e and g, statistical significance was assessed using Welch’s t-test. Error bars represent SD.

The question of whether pathogenic mutations cause a functional deficiency of FUS or result in a toxic gain of function is critical to determining a therapeutic approach to ALS-FUS, by either silencing or restoration of lost function. FUS plays a critical role in development, as shown by the perinatal lethality of the elimination of FUS in knockout mice (KO/KO)^24^. However, in previous studies^12^, we showed that postnatal FUS deficiency is insufficient to cause MN degeneration, and we concluded, based on indirect evidence, that the toxicity of mutant FUS was a consequence of a gain of function. Here, using the P517L and Δ14 mutant FUS knock-in alleles in combination with the wild-type and knockout FUS alleles, we took a genetic approach to assess the effect of these mutations on FUS function and the effect of gene dosage on the degenerative MN phenotype that we observed in vivo in these knock-in animals. In a series of experiments, we crossed mice carrying *Fus* wild-type, mutant knock-in (P517L or Δ14) and knockout alleles to generate homozygous, heterozygous and hemizygous animals for each *Fus* allele. These experiments show that P517L and Δ14 mutant proteins retain partial FUS function and incompletely rescue the FUS knockout phenotype (Fig. 2a,b, Extended Data Fig. 3a,b and Table 1).

**Table 1:** Summary of phenotypes of mutant FUS knock-in mouse lines

Genotype	Birth weight^a^	Suvival	MN loss	Muscle innervation	Other phenotypes
WT/WT	1.40 ± 0.01 g *n* = 183	Normal	Normal	Normal	NA
P517L/WT	1.37 ± 0.01 g *n* = 157	~90% survive to weaning	Onset at 1.5 years	TA denervation onset at 1 year	Malocclusion and rectal prolapse (all rare)
Δ14/WT	1.36 ± 0.01 g *n* = 173	~90% survive to weaning	Onset at 1.5 years	TA denervation onset at 1 year	Malocclusion and rectal prolapse (all rare)
WT/KO	1.34 ± 0.02 g *n* = 95	Normal	Normal	Normal	No known phenotype
KO/KO	1.00 ± 0.02 g *n* = 26	Perinatal lethal	Normal	Normal at birth	Hicks et al., 2000
P517L/KO	1.15 ± 0.02 g *n* = 14	21 ± 12 d	NA	Normal at birth	Malocclusion, rectal prolapse and seizures
Δ14/KO	1.02 ± 0.02 g *n* = 13	Perinatal lethal; max survival ~1 week	NA	NA	NA
P517L/P517L	0.98 ± 0.02 g *n* = 26	Perinatal lethal	Normal at birth	Normal at birth	NA
Δ14/Δ14	1.09 ± 0.01 g *n* = 50	~20% survive to weaning	Onset at 1 year	NA	Malocclusion, rectal prolapse and seizures
P517L/Δ14	1.10 ± 0.02 g *n* = 44	Perinatal lethal; max survival ~3 weeks	NA	NA	NA
MN-Δ14/WT: c14/WT; ChAT-Cre	NA	NA	Onset at 1.5 years	NA	NA
MN-Δ14/Δ14: c14/c14; ChAT-Cre c14/Δ14; ChAT-Cre	NA	NA	Onset at 1 year	NA	NA
MN-P517L/Δ14: P517L/c14; ChAT-Cre	1.42 ± 0.03 g *n* = 28	~90% survive to weaning	Onset at 4 months	TA denervation onset at 4 months	Malocclusion, rectal prolapse and seizures (all rare)

^a^Mean ± s.e.m. *n* represents number of individual animals. NA, not available.

The partial loss of function caused by the P517L and Δ14 mutations could selectively affect specific activities of FUS or incompletely reduce all protein activities. In the latter case, two copies of the mutant allele would be expected to restore more of the normal FUS activity and further improve the knockout phenotype. Indeed, this is the case for Δ14, as the homozygous mutants (Δ14/Δ14) showed an improved survival compared to the hemizygous mutants (Δ14/KO) despite similar birth weights (Fig. 2a,b, Extended Data Fig. 3a and Table 1). By contrast, P517L/P517L animals were born at lower than expected Mendelian ratios and were smaller than P517L/KO animals, and none survived more than several hours after birth (Fig. 2a,b, Extended Data Fig. 3a,b and Table 1). Surprisingly, P517L/Δ14 animals also had reduced survival compared to the P517L/KO animals (Fig. 2a,b and Table 1). The exacerbation of the low birth weight and poor survival phenotype of the P517L/KO mutants by additional P517L protein (in P517L/P517L homozygous animals) or Δ14 protein (in P517L/Δ14 compound heterozygous mutants) shows dose-dependent gain-of-function toxicity of P517L and Δ14 mutant FUS. The increased severity of the P517L/P517L phenotype compared to the P517L/Δ14 phenotype suggests that P517L is more toxic than Δ14 mutant FUS, and this conclusion is further supported by decreased long-term survival of successfully weaned P517L/WT animals relative to Δ14/WT animals (Extended Data Fig. 3c). Notably, although each of these mutant alleles is sufficient to cause age-dependent, selective MN degeneration in the heterozygous mice, the decreased perinatal survival of P517L/P517L, P517L/Δ14 and Δ14/Δ14 animals appears to be unrelated to MN degeneration, as no denervation or MN loss was detected in these animals at postnatal day 0 (P0) (Extended Data Fig. 3d–g and Table 1).

**Extended Data Fig. 4:**
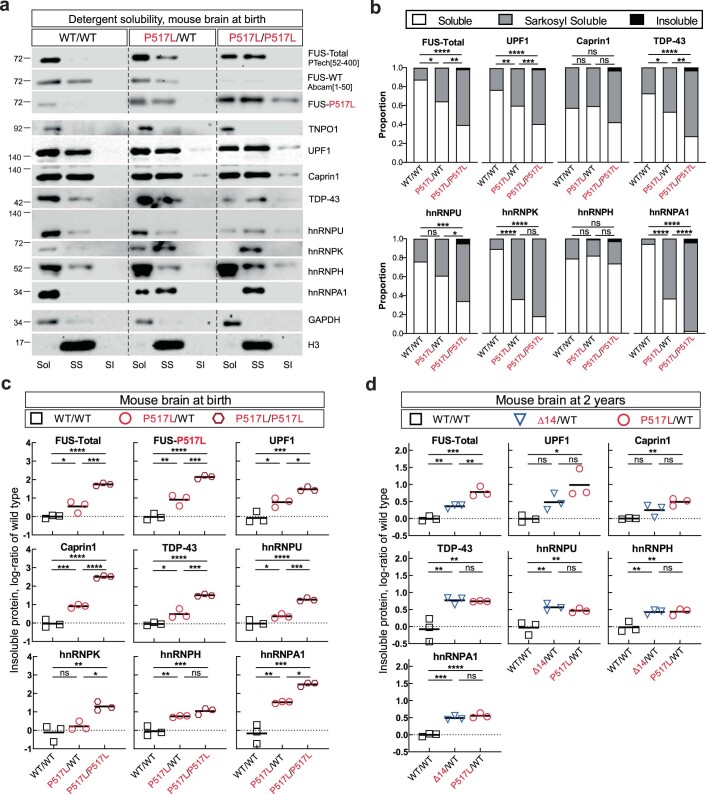
Detergent insolubility of mutant FUS and related RNA-binding proteins. **(a)** Immunoblot of sarkosyl solubility fractionation of brain from newborn FUS WT/WT, P517L/WT, and P517L/P517L mice. Sol = soluble (in hypotonic buffer), SS = sarkosyl soluble (in 1% sarkosyl and high salt), and SI = sarkosyl insoluble fractions. **(b)** Quantitation of protein in Sol (Soluble), SS (Sarkosyl Soluble), and SI (Insoluble) fractions. *P < 0.05, **P < 0.01 and ***P < 0.001 comparing Soluble versus the sum of Sarkosyl Soluble and Insoluble fractions using one-way ANOVA with Tukey’s post hoc test. N = 3 animals per group. **(c)** Quantitation of protein in sarkosyl insoluble fractions from newborn animals shown in Fig. 3c, expressed as log-ratio of wild type. **(d)** Quantitation of protein in sarkosyl insoluble fractions from 2-year-old animals shown in Fig. 3d, expressed as log-ratio of wild type. For c and d, *P < 0.05, **P < 0.01 and ***P < 0.001 using one-way ANOVA with Tukey´s post hoc test. N = 3 animals per genotype. Source data

Mutant FUS toxicity might relate to its irreversible incorporation into cytoplasmic inclusions or detergent-insoluble protein aggregates, resulting in aberrant RNA processing^25,26^. Although we do not observe obvious cytoplasmic FUS inclusions in MNs by immunofluorescent staining (Extended Data Fig. 2c,d,h), biochemical fractionation of brains of P0 animals reveals increased partitioning of FUS from the soluble to the detergent-soluble and detergent-insoluble fractions in P517L/WT and P517L/P517L animals compared to wild-type controls (Extended Data Fig. 4a,b). Indeed, side-by-side analysis of only the detergent-insoluble fractions from individual brains fractionated in the same experiments clearly shows the increase in detergent-insoluble FUS in P517L/WT relative to wild-type animals and a further accumulation of insoluble FUS in P517L/P517L relative to P517L/WT animals (Fig. 2c and Extended Data Fig. 4c). Given the insolubility of a broad range of related RBPs that we observed in the brain and spinal cord of patients with ALS/FTD carrying the *C9orf72* expansion mutation and in sporadic ALS^27^, we looked in the P517L/WT and P517L/P517L animals to see if FUS insolubility was associated with a similar increase in insoluble RBPs. We found increased partitioning of TDP-43 as well as hnRNP A1, hnRNP H, hnRNP U and other RBPs from the soluble to the detergent-soluble and detergent-insoluble fractions in newborn and 2-year-old P517L/WT animals (Fig. 2c,d and Extended Data Fig. 4c,d). To validate that the RBP insolubility in the FUS mutant mice reflects pathological changes in ALS-FUS, we performed the same fractionation on a postmortem brain sample from an ALS-FUS^P525L^ patient and showed insolubility of both total and P525L mutant FUS that was not seen in the age-matched, non-neurological control brain (Fig. 2e). Moreover, we also observed increased partitioning of TDP-43, hnRNP A1, hnRNP H, hnRNP U and other RBPs from the soluble to the detergent-insoluble fractions of the FUS^P525L^ ALS brain, showing that, in patients with ALS-FUS, as in the mutant mice, FUS drives the insolubility of related RBPs, including TDP-43 (Fig. 2f).

To test the functional significance of this increased insolubility of FUS and other RBPs, we looked in the mutant animals for evidence of aberrant splicing of their target pre-mRNAs, similar to that which we described previously in brain and spinal cord tissue from C9orf72-positive and gene-negative patients with ALS/FTD^27^. As in those cases, RBP insolubility in the Δ14/WT and P517L/WT animals was also associated with alteration in splicing. For example, analysis of two targets of hnRNP H showed decreased inclusion of *ATXN2* exon 24 and increased inclusion of *hnRNPDL* exon 8—consistent with decreased hnRNP H activity—in Δ14/WT and P517L/WT animals compared to wild-type controls (Fig. 2g). P517L/WT showed a greater magnitude of detergent insolubility and splicing alterations compared to Δ14/WT animals (Fig. 2d,g and Extended Data Fig. 4d), once again suggesting increased toxicity of P517L over Δ14.

Taken together, these experiments reveal that normal FUS function is essential for perinatal mouse survival and that P517L and, to a lesser extent, Δ14 mutant FUS protein retain sufficient activity for a single copy of either allele to rescue the knockout phenotype, albeit partially. However, when expressed at higher levels in homozygous or compound heterozygous animals, mutant FUS protein is toxic in a dose-dependent manner, decreasing mutant survival. This toxicity is associated with increased insolubility of FUS, TDP-43 and related RBPs, many of which have been independently implicated in the pathogenesis of ALS. This insolubility leads to the consequent loss of function of these regulatory proteins and to the aberrant processing of their respective target RNAs.

### Cell-autonomous expression of FUS Δ14 accelerates MN degeneration

**Fig. 3:**
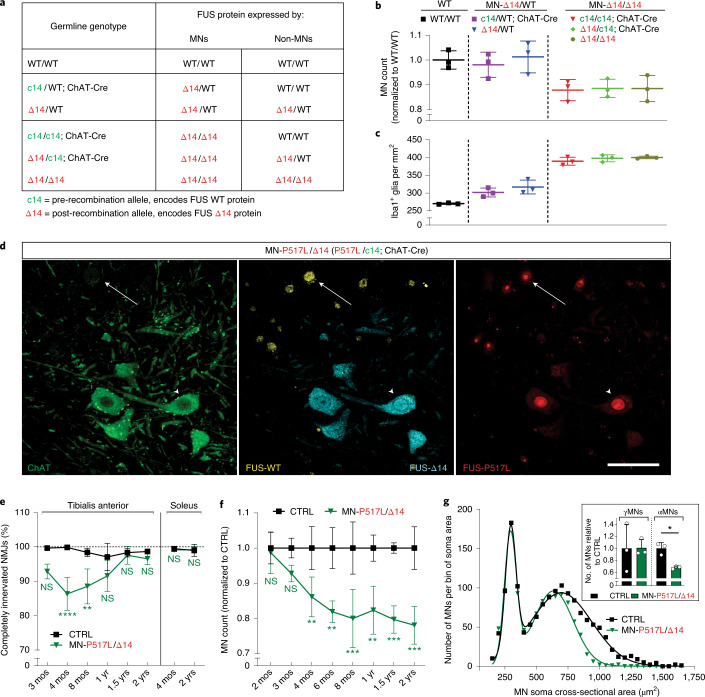
Increased dosage of mutant FUS in MNs accelerates selective MN degeneration. **a**, Table relating the indicated mouse genotypes to WT and Δ14 FUS protein expression in MN and non-MN cells. **b**, Numbers of ChAT-positive MNs at lumbar levels 4 and 5 in 1-year-old WT/WT (left), MN-Δ14/WT (middle, c14/WT; ChAT-Cre and Δ14/WT) and MN-Δ14/Δ14 (right, c14/c14; ChAT-Cre, Δ14/c14; ChAT-Cre and Δ14/Δ14) animals normalized to the wild-type controls. Each of the MN-Δ14/Δ14 genotypes shows fewer lumbar MNs in comparison to either of MN-Δ14/WT genotypes or WT/WT group, using one-way ANOVA followed by Fisher’s least significant difference. Data are shown as mean ± s.d. *n* = 3 animals per genotype. **c**, Density of Iba1-positive microglial cells at lumbar levels 4 and 5 in 1-year-old WT/WT (left, black), MN-Δ14/WT (middle, c14/WT; ChAT-Cre and Δ14/WT) and MN-Δ14/Δ14 (right, c14/c14; ChAT-Cre, Δ14/c14; ChAT-Cre and Δ14/Δ14) animals. Each of the MN-Δ14/WT genotypes show an increase in microglial density in comparison to WT/WT animals. Each of the MN-Δ14/Δ14 genotypes has increased microglial density in comparison to either of MN-Δ14/WT genotypes or WT/WT group. Statistical significance was determined by one-way ANOVA with Tukey’s post hoc test. Data are shown as mean ± s.d. *n* = 3 animals per genotype. **d**, Immunostaining of lumbar spinal cord sections of adult MN-P517L/Δ14 (P517L/c14; ChAT-Cre) animals using anti-ChAT (green), anti-FUS-WT (Abcam[1-50], yellow), anti-FUS-Δ14 (cyan) and mouse monoclonal anti-FUS-P517L (red) antibodies. Note anti-FUS-P517L staining in all cells, anti-FUS-Δ14 staining only in ChAT-positive cells and anti-FUS-WT staining only in ChAT-negative cells. Scale bar, 100 µm. **e**, Percentage of completely innervated NMJs (that is, not partially or completely denervated) in TA (left) and soleus (right) muscles of CTRL (FUS WT-expressing control, black) and MN-P517L/Δ14 (P517L/c14; ChAT-Cre, green) animals. **f**, MN numbers at lumbar level 4 and 5 of CTRL (FUS WT-expressing control, black) and MN-P517L/Δ14 (P517L/c14; ChAT-Cre, green) animals normalized to the controls. **g**, Histogram of MN soma cross-sectional areas of CTRL (FUS WT-expressing control, black) and MN-P517L/Δ14 (P517L/c14; ChAT-Cre, green) animals. MNs with soma area ≥475 µm^2^ were classified as alpha-MNs, and MNs with soma area <475 µm^2^ were classified as gamma-MNs. Inset shows the numbers of ChAT-positive gamma-MNs (left) or alpha-MNs (right) at lumbar levels 4 and 5 in 2-year-old CTRL (FUS WT-expressing control, black) and MN-P517L/Δ14 (P517L/c14; ChAT-Cre, green) animals normalized to the controls. *P < 0.05, **P < 0.01 and ***P < 0.001, using Welch’s *t*-test. Data are shown as mean ± s.d. *n* = 3 animals per group. For **e** and **f**, *P < 0.05, **P < 0.01 and ***P < 0.001, using two-way ANOVA with Sidak´s post hoc test. Data are shown as mean ± s.d. *n* = 3 animals per group. CTRL, littermate c14/WT and/or c14/c14 animals depending on the breeding scheme.

Consistent with their highly pathogenic nature in patients with ALS-FUS, a single copy of the P517L or Δ14 allele is sufficient to cause progressive MN degeneration in the lifetime of heterozygous animals, providing a faithful mouse model of disease. As we showed, increasing the dose of mutant FUS in the P517L/P517L, P517L/Δ14 and Δ14/Δ14 mice results in increased toxicity, leading to perinatal lethality, seemingly due to widespread toxicity of mutant FUS in embryonic development. To avoid this developmental toxicity and restrict the dose-dependent effects of mutant FUS to MNs, we generated a Δ14/c14 mutant that carries the Δ14 allele and the conditional FUS c14 allele, which encodes wild-type FUS but is transformed into the Δ14 allele after Cre-dependent recombination (Fig. 1b). We used the ChAT-Cre allele^28^ to activate FUS c14 selectively in cholinergic cells so that only MNs are Δ14/Δ14 homozygous in this animal, whereas non-cholinergic cells remain Δ14/c14 and express only a single copy of the Δ14 mutant allele. To account for potential non-MN autonomous mutant FUS-dependent effects, we generated additional genotypes that are MN heterozygous or homozygous in a WT or Δ14/WT background (Fig. 3a).

**Extended Data Fig. 5:**
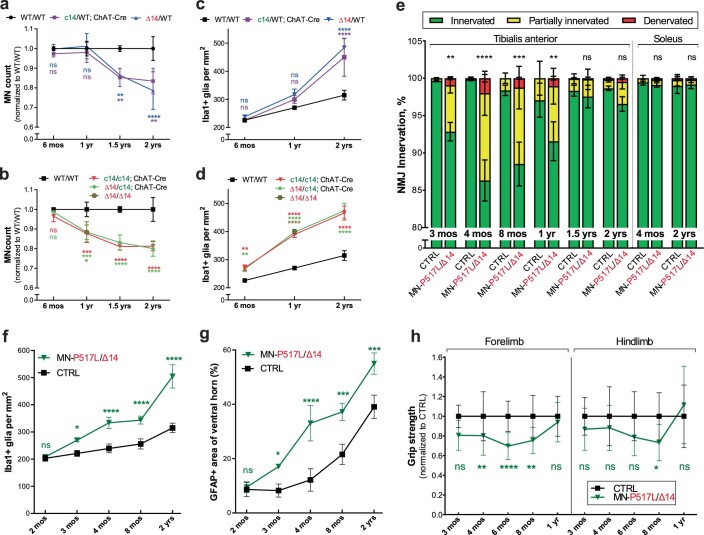
Conditional c14 expression: NMJ denervation, selective motor neuron loss, gliosis and grip strength. **(a)** Numbers of ChAT positive motor neurons at lumbar levels 4 and 5 in WT/WT (black) and MN-Δ14/WT (c14/WT; ChAT-Cre and Δ14/WT, purple and blue respectively) animals normalized to the wild type controls. (**b**) Numbers of ChAT positive motor neurons at lumbar levels 4 and 5 in WT/WT (black) and MN-Δ14/Δ14 (c14/c14; ChAT-Cre, Δ14/c14; ChAT-Cre, and Δ14/Δ14, red, green, and brown respectively) animals normalized to the controls. **(c)** Density of Iba1-positive microglial cells at lumbar levels 4 and 5 in WT/WT (black) and MN-Δ14/WT (c14/WT; ChAT-Cre and Δ14/WT, purple and blue respectively) animals. **(d)** Density of Iba1-positive microglial cells at lumbar levels 4 and 5 in WT/WT (black) and MN-Δ14/Δ14 (c14/c14; ChAT-Cre, Δ14/c14; ChAT-Cre, and Δ14/Δ14, red, green, and brown respectively) animals. **(e)** Percentages of NMJs classified as fully innervated (green), partially denervated (yellow), and fully denervated (red) in the tibialis anterior (left) and soleus (right) muscles in CTRL (FUS WT-expressing control) and MN-P517L/Δ14 (FUS P517L/c14; ChAT-Cre) animals. *P < 0.05, **P < 0.01 and ***P < 0.001 indicates significant differences in fully denervated NMJs using two-way ANOVA with Sidak´s post hoc test. Data shown as mean ± SD. N = 3 animals per group. **(f)** Density of Iba1-positive microglial cells at lumbar levels 4 and 5 in CTRL (FUS WT-expressing control, black) and MN-P517L/Δ14 (FUS P517L/c14; ChAT-Cre, green) animals. **(g)** Percentage of cross-sectional area that is GFAP-positive at lumbar levels 4 and 5 in CTRL (FUS WT-expressing control, black) and MN-P517L/Δ14 (FUS P517L/c14; ChAT-Cre, green) animals. **(h)** Normalized grip strength of forelimbs (left) and hindlimbs (right) in CTRL (FUS WT-expressing control, black) and MN-P517L/Δ14 (P517L/c14; ChAT-Cre, green) animals normalized to the controls. *P < 0.05, **P < 0.01 and ***P < 0.001 using two-way ANOVA with Sidak´s post hoc test. Data shown as mean ± SD. N = 5–28 per group at different time points. For a-d, *P < 0.05, **P < 0.01 and ***P < 0.001 using two-way ANOVA with Tukey´s post hoc test. Data shown as mean ± SD. N = 3 animals per genotype. For f and g, *P < 0.05, **P < 0.01 and ***P < 0.001 using two-way ANOVA with Sidak´s post hoc test. Data shown as mean ± SD. N = 3 animals per group. CTRL=littermate c14/WT and/or c14/c14 animals depending on the breeding scheme.

To test whether an MN-restricted ‘double dose’ of mutant FUS accelerates ALS-related pathology, we compared mice expressing either one or two copies of mutant FUS in MNs at 1 year of age—a time point at which MN loss is not yet detected in heterozygous mutant FUS animals (Fig. 1c). As expected, no MN loss was observed at 1 year in ‘MN-Δ14/WT’ mice expressing one copy of a Δ14 allele in MNs (Δ14/WT and c14/WT; ChAT-Cre) (Fig. 3a,b). By contrast, ‘MN-Δ14/Δ14’ mice expressing two Δ14 alleles in MNs (Δ14/Δ14, Δ14/c14; ChAT-Cre and c14/c14; ChAT-Cre) all showed a similar level of approximately 10% MN loss, indicating acceleration of MN degeneration by the expression of the second Δ14 allele in MNs (Fig. 3a,b). Similarly, an increase in the Iba1 microglial marker staining was observed in MN-Δ14/Δ14 animals relative to MN-Δ14/WT mice, showing that expression of the second Δ14 allele in MNs accelerates microgliosis (Fig. 3a,c). Time course analysis revealed that microgliosis is first detected at 6 months, and MN degeneration is first detected at 1 year, in MN-Δ14/Δ14 mice, showing that expression of an additional Δ14 allele in MNs of MN-Δ14/Δ14 mice accelerates the onset of both microgliosis and MN loss by 6 months compared to MN-Δ14/WT animals (Extended Data Fig. 5a–d).

The conditional expression of Δ14 mutant FUS in MNs showed that the dosage of mutant protein determines the age of onset of MN degeneration in vivo. These experiments also revealed the cell-autonomous nature of this dose-dependent toxicity. The timing of microgliosis and subsequent MN loss in Δ14/WT animals was identical to that of c14/WT; ChAT-Cre mutants. The same was true in Δ14/Δ14, Δ14/c14; ChAT-Cre and c14/c14; ChAT-Cre mice, indicating that the MN degenerative phenotype is determined by the level of mutant Δ14 FUS expression specifically in MNs, without measurable contribution of mutant FUS expression in other cell types. Furthermore, these data suggest that microgliosis—and other possible, cell-non-autonomous contributors to MN degeneration—is a pathological response to mutant FUS-dependent MN pathology, as it occurs independently of mutant FUS expression in the responding microglia (Extended Data Fig. 5a–d).

Because our previous experiments suggested that mutant P517L is more pathogenic than Δ14, we suspected that co-expression of these two mutant alleles in MNs would further accelerate disease. We, therefore, generated P517L/c14; ChAT-Cre (‘MN-P517L/Δ14’) conditional compound heterozygote mutants that express both P517L and Δ14 mutant FUS only in MNs, on an otherwise P517L- and WT-expressing background. Immunofluorescent staining of spinal cords confirmed that, in MN-P517L/Δ14 mice, MNs express P517L and Δ14 (but not WT) FUS, whereas non-cholinergic cells express P517L and WT (but not Δ14) FUS (Fig. 3d). As predicted, addition of the more pathogenic P517L allele led to NMJ denervation, microgliosis and astrogliosis at 3 months of age and MN degeneration at 4 months in MN-P517L/Δ14 mice (Fig. 3e,f and Extended Data Fig. 5f,g)—earlier than the corresponding changes in MN-Δ14/Δ14 animals (Extended Data Fig. 5b,d).

Despite this earlier onset, MN loss in MN-P517L/Δ14 mutants was limited to the same selectively vulnerable pools as in the heterozygous P517L/WT and Δ14/WT mice. The vulnerable TA muscle showed NMJ denervation, whereas the soleus muscle remained unaffected (Fig. 3e and Extended Data Fig. 5e). Here again, MN loss was limited to the large-diameter (alpha) MNs (Fig. 3g) and ultimately plateaued at around 20% (Fig. 3f), approximately the same level as in heterozygous P517L/WT and Δ14/WT mice (Fig. 1c), suggesting depletion of the same subset of vulnerable MNs. Of note, denervation of the TA recovered in time: re-innervation of NMJs (Fig. 3e), along with functional recovery from mild deficiencies in grip strength (Extended Data Fig. 5h), occurred by 1 year of age.

**Fig. 4:**
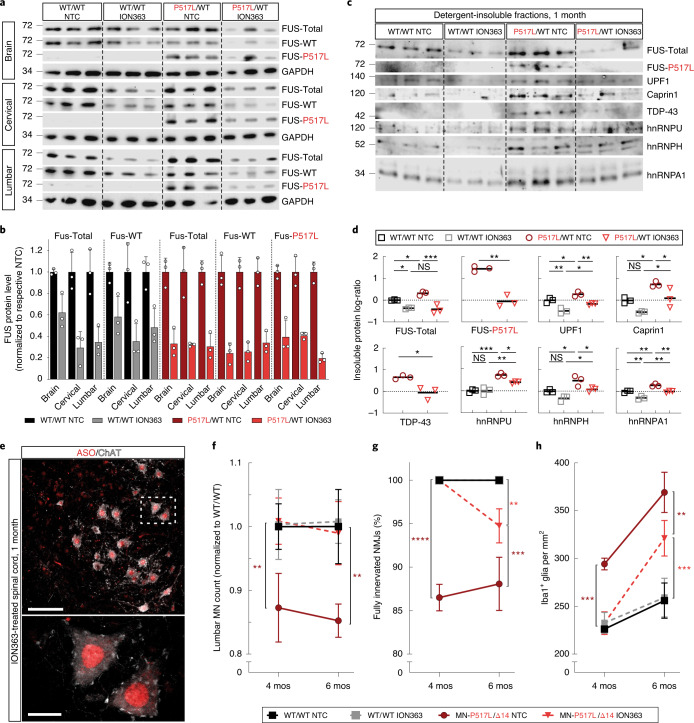
Efficacy of the FUS ASO ION363 in FUS-ALS knock-in mice. **a**, Immunoblot probed with anti-FUS antibodies of brain and cervical or lumbar spinal cord protein lysates of 1-month-old WT/WT and P517L/WT animals treated with NTC or anti-*FUS* oligonucleotide (ION363). Each band in a row represents a separate animal. **b**, Quantitation of total, WT and P517L FUS protein levels shown in **a**. Welch’s *t*-test was used for comparisons of NTC versus ION363 within each genotype and neuroanatomical region. Data are shown as mean ± s.d. *n* = 3 animals per group. **c**, Immunoblot of brain sarkosyl-insoluble fractions of 1-month-old WT/WT and P517L/WT animals treated with NTC or anti-*FUS* oligonucleotide (ION363). Each lane represents a separate animal. **d**, Quantitation of protein in sarkosyl-insoluble fractions shown in **c**, expressed as log-ratio of P517L/WT ION363 for FUS-P517L and TDP-43 and as log-ratio of WT/WT NTC for all others. *P < 0.05, **P < 0.01 and ***P < 0.001, using one-way ANOVA with Tukey´s post hoc test for comparison of four groups and Welch’s *t*-test for two groups. *n* = 3 animals per group. Individual values and means are shown. **e**, Immunostaining of lumbar spinal cord sections of a 1-month-old ION363-treated animal with anti-ASO (red) and anti-ChAT (white) antibodies showing broad distribution of ION363 to MNs and other cells and predominantly nuclear localization. Scale bar, 100 µm at ×20 and 20 µm at ×100. **f**, Numbers of ChAT-positive MNs at lumbar levels 4 and 5 in NTC-treated FUS WT-expressing CTRL (black), ION363-treated FUS WT-expressing CTRL (gray), NTC-treated MN-P517L/Δ14 (dark red) and ION363-treated MN-P517L/Δ14 (light red) animals normalized to the NTC-treated FUS WT-expressing CTRL. **g**, Percentage of fully innervated NMJs in the TA muscles in NTC-treated FUS WT-expressing CTRL (black), ION363-treated FUS WT-expressing CTRL (gray), NTC-treated MN-P517L/Δ14 (dark red) and ION363-treated MN-P517L/Δ14 (light red) animals. **h**, Density of Iba1-positive microglial cells at lumbar levels 4 and 5 in NTC-treated FUS WT-expressing CTRL (black), ION363-treated FUS WT-expressing CTRL (gray), NTC-treated MN-P517L/Δ14 (dark red) and ION363-treated MN-P517L/Δ14 (light red) animals. For **f**–**h**, *P < 0.05, **P < 0.01 and ***P < 0.001, using two-way ANOVA with Tukey’s post hoc test. Data are shown as mean ± s.d. *n* = 3 animals per group. Source data

Overall, the data presented in Fig. 4 show cell-autonomous toxicity of mutant FUS—as conditional expression of the Δ14 allele selectively in MNs is sufficient for MN degeneration and gliosis. This toxicity is dose dependent. Expression of two mutant FUS alleles in MNs accelerates the onset of NMJ denervation, MN loss and gliosis, with MN-P517L/Δ14 mice showing a particularly early disease onset. The selective degeneration of the same subpopulation of ALS-vulnerable MNs in MN-P517L/Δ14 mutants as in P517L/WT and Δ14/WT animals support the conclusion that MN-P517L/Δ14 mice are a disease-relevant model of early-onset ALS.

### Antisense silencing of FUS in mice prevents MN degeneration

Our studies of the consequences of mutant FUS in this series of knock-in mice show a dose-dependent toxicity of mutant FUS that results in progressive degeneration of a select subpopulation of spinal MNs. This, together with our previous study using a conditional FUS knockout allele that showed that postnatal FUS deficiency in vivo does not result in MN degeneration^12^, suggests that lowering levels of FUS protein in vulnerable neurons could be an effective strategy to treat patients with ALS-FUS. To test this idea, we used ION363, an ASO directed against the 6th intron of the FUS transcript that was developed by Ionis Pharmaceuticals to silence FUS in a non-allele-specific manner. ION363 was chosen from a series of candidate antisense therapeutics because the target intronic sequence is conserved between mouse and human, which would allow us to translate our findings in the FUS knock-in animals to clinical studies of patients with ALS-FUS.

First, to evaluate the efficacy of ION363 in lowering FUS levels in vivo in the mouse, we administered a single 20-μg dose of ION363 via ICV injection to newborn (postnatal day 1 (P1)) wild-type and P517/WT animals. Control animals received an identical injection of a non-targeted control (NTC) ASO. Western immunoblot analysis of brain and spinal cord of 1-month-old ASO-treated animals showed an overall decrease in both wild-type and mutant FUS protein to approximately 20–50% of the levels observed in matched controls (Fig. 4a,b).

We next analyzed whether detergent insolubility of RBPs, which is the biochemical signature associated with FUS toxicity (Fig. 2c–f), is affected by ION363 treatment. Indeed, a decrease in the levels of TDP-43, hnRNP A1, hnRNP U and UPF1 was observed in the detergent-insoluble fractions from ION363-treated versus NTC P517L/WT mice (Fig. 4c,d). Moreover, the ION363-induced decrease in wild-type FUS protein in wild-type mice also caused decreased partitioning of UPF1 and hnRNP A1 into the detergent-insoluble fractions (Fig. 4c,d), suggesting that wild-type FUS-dependent partitioning of some RBPs into detergent-insoluble subcellular compartments at a low level might reflect normal physiology.

**Extended Data Fig. 6:**
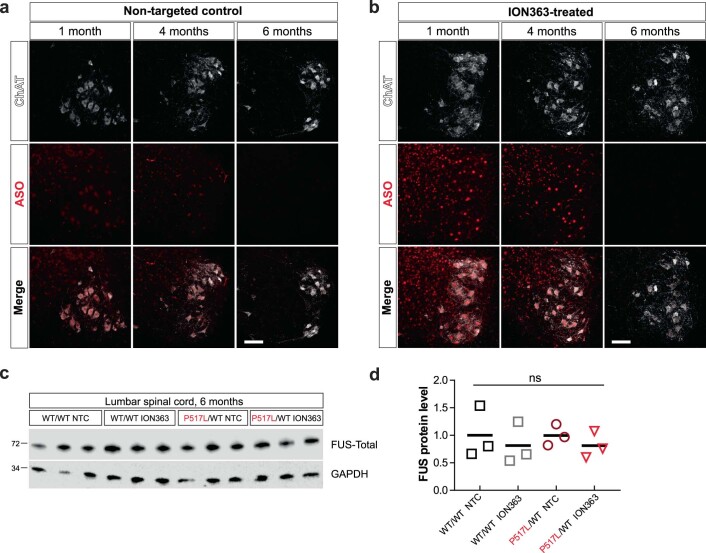
FUS silencing by ION363 in FUS-ALS mice. **(a)** Representative immunostaining of lumbar spinal cord sections from NTC-treated animals with an anti-ASO antibody. The staining shows widespread nuclear and cytoplasmic distribution at 1 and 4 months of age that disappears at 6 months. Scale bar=100 µm. **(b)** Representative immunostaining of lumbar spinal cord sections from FUS ASO-treated animals with an anti-ASO antibody. The staining shows widespread nuclear distribution at 1 and 4 months of age that disappears at 6 months. Scale bar=100 µm. **(c)** Immunoblot of lumbar spinal cord protein from 6-month-old WT/WT and P517L/WT animals treated with non-targeted control (NTC) or anti-*FUS* oligonucleotide (ION363). Each lane represents a separate animal. (**d**) Quantitation of FUS protein levels in lumbar spinal cord lysates from 6-month-old animals shown in (c). One-way ANOVA was performed to assess the differences in FUS levels. N = 3 animals per group. Source data

Immunostaining of spinal cords with an antibody^29^ to the modified backbone of the ION363 and NTC ASOs showed robust predominantly nuclear signal in all cells, including MNs (Fig. 4e and Extended Data Fig. 6a,b) at 1 and 4 months of age. However, by 6 months of age, analysis of anti-ASO immunostaining showed near background levels of signal for both ION363 and the NTC ASO (Extended Data Fig. 6a,b). The observed decrease in ION363 levels at 6 months of age was associated with a corresponding increase in FUS levels (Extended Data Fig. 6c,d).

The model of disease that we propose for ALS-FUS based on our animal studies predicts that the decrease in FUS protein levels—and associated decrease in detergent insolubility of FUS and related RBPs—caused by ION363 would mitigate the MN phenotype in the FUS mutant mice. To test this prediction, we injected newborn (P1) MN-P517L/Δ14 and wild-type mice with a single ICV dose of 20 μg of ION363 or the NTC ASO and quantified MN loss in all experimental animals at 4 and 6 months of age. At 4 months, the NTC-injected MN-P517L/Δ14 mice lost an average of 12% of all lumbar MNs (consistent with untreated MN-P517L/Δ14 mice; Fig. 3f), but no MN loss was observed in ION363-treated MN-P517L/Δ14 mice (Fig. 4f). Similarly, injections of ION363, but not NTC, completely prevented muscle denervation and microgliosis, both of which precede MN loss, in the MN-P517L/Δ14 mutants at 4 months (Fig. 4g,h). At 6 months of age, when ION363 concentration decreases and FUS levels normalize (Extended Data Fig. 6d), muscle denervation and microgliosis increased in ION363-treated MN-P517L/Δ14 mice to levels intermediate between those of NTC-injected MN-P517L/Δ14 and wild-type mice, suggesting the delayed onset of the disease pathology in this model. Nevertheless, no MN degeneration was observed in ION363-treated MN-P517L/Δ14 mice at 6 months after treatment (Fig. 4f).

These pre-clinical data show that a single perinatal injection of ION363 results in a robust and persistent decrease in FUS protein levels for more than 4 months, and that, while the ASO perdures in the spinal cord, the onset of gliosis is delayed and MN loss is prevented, showing the therapeutic potential of FUS silencing—and of this specific FUS-ASO—in the treatment of patients with ALS-FUS.

### Therapeutic silencing of FUS with an ASO in a patient with ALS-FUS

**Fig. 5:**
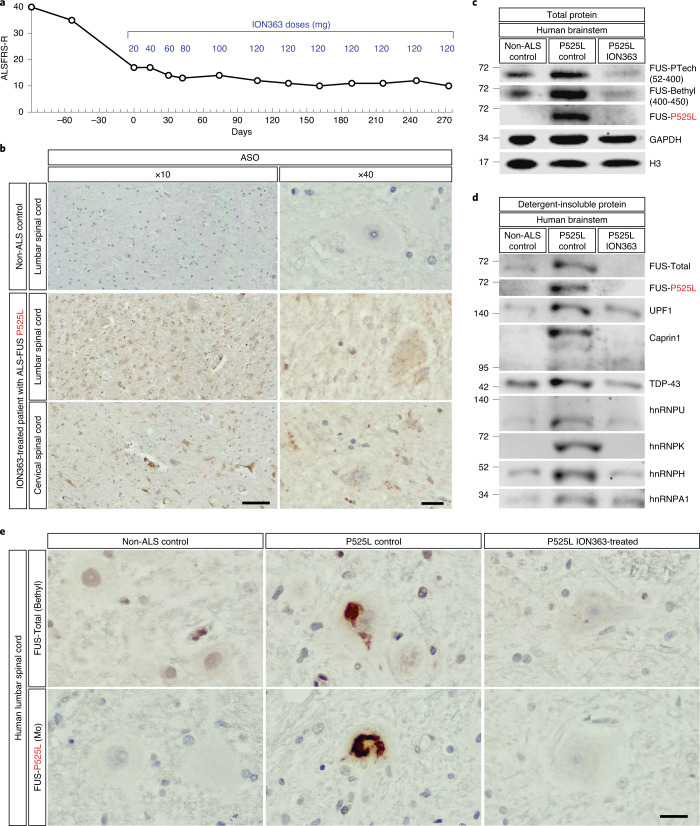
First-in-human treatment with ION363 silences FUS expression, decreases FUS pathology and reverses insolubility of RBPs in an ALS-FUS^P525L^ patient. **a**, Timeline of patient J.H.’s ALSFRS-R scores relative to ION363 infusions. Treatment started with 20 mg of ION363 on day 0 and escalated to 120 mg per dose. A total of 12 infusions was administered. Numbers above open circles indicate ION363 doses in milligrams. **b**, Anti-ASO immunohistochemical staining of formalin-fixed, paraffin-embedded (FFPE) sections of lumbar spinal cord from a non-ALS control (top) and lumbar (middle) and cervical (bottom) spinal cord from ION363-treated ALS-FUS^P525L^ patient. Scale bars, 100 µm at ×10 and 20 µm at ×40. **c**, Immunoblot of brainstem tissue from a non-ALS control, an ALS-FUS^P525L^ control patient and an ION363-treated patient with ALS-FUS^P525L^ probed with antibodies against non-overlapping epitopes of FUS. **d**, Immunoblot of equal volumes of sarkosyl-insoluble fractions from brainstem tissue from a non-ALS control, an ALS-FUS^P525L^ control patient and an ION363-treated patient with ALS-FUS^P525L^ probed with antibodies against total FUS (FUS-Proteintech[52-400]), FUS-P525L and other RBPs. **e**, Immunohistochemical staining of FFPE sections from lumbar spinal cord of a non-ALS control, an ALS-FUS^P525L^ control patient and an ION363-treated patient with ALS-FUS^P525L^ with an antibody against total FUS (FUS-Bethyl[400-450]; top) and monoclonal (Mo) P525L-specific antibody reactive to FUS aggregates (bottom). Scale bar, 20 µm. Source data

ION363 targets an intronic sequence identical in mouse and human and, therefore, represents a promising therapeutic strategy to decrease FUS toxicity in patients with ALS. With this rationale, we successfully submitted a single-patient, individual expanded access (‘compassionate use’) Investigational New Drug application to the US Food and Drug Administration (FDA) to treat a patient with ALS (J.H.) with a FUS^P525L^ mutation^30^. At symptom onset, J.H. was a 25-year-old woman whose identical twin sister had died of complications of ALS caused by this mutation. As is typical of juvenile-onset ALS-FUS^P525L^ cases, J.H. progressed rapidly after disease onset, initially losing more than five points per month on the 48-point ALS Functional Rating Scale-Revised (ALS-FRS-R)^31^. Treatment with ION363 began more than 6 months after clinical onset, by which time J.H.’s disease had progressed to the point that J.H. was no longer ambulatory and required non-invasive ventilatory support (ALS-FRS-R score of 17). Under this experimental, first-in-human protocol, J.H. received ascending IT doses of ION363, starting with 20 mg up to a maximum monthly dose of 120 mg IT for a total of 12 infusions over 10 months (Fig. 5a). J.H. tolerated the experimental protocol well, without any serious adverse events. Through the course of therapy, the rate of decline in the ALS-FRS-R score slowed substantially (Fig. 5a), but, consistent with the natural history of ALS-FUS^P525L^, J.H. developed worsening of ventilatory and bulbar dysfunction and died of complications of the disease 18 months after disease onset, nearly 1 year after initial treatment with ION363. A full autopsy including neuropathological examination was performed, and brain and spinal cord tissue was banked for analysis.

**Extended Data Fig. 7:**
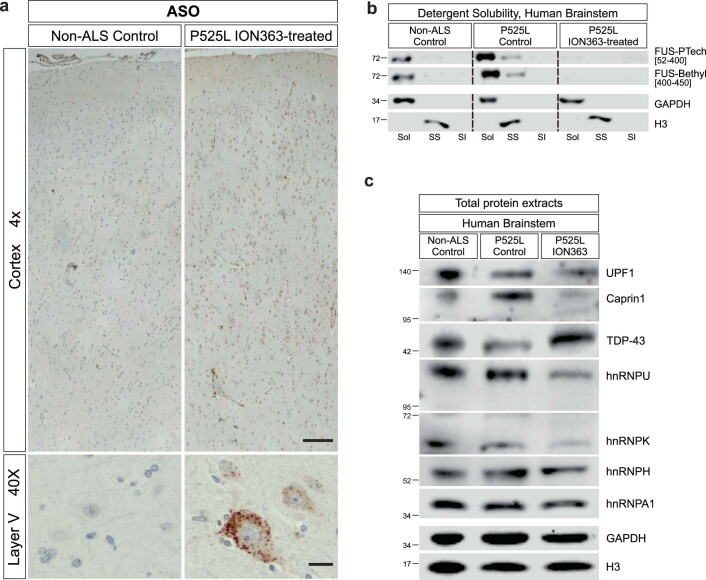
Cortical distribution of ION363 and its effect on total protein levels of RBPs. (**a**) Anti-ASO immunohistochemical staining of FFPE sections of motor cortex (BA4) from a non-ALS control (left) and ION363-treated ALS-FUS^P525L^ patient (right). Scale bar=200 µm at 4x and 20 µm at 40x. (**b**) Immunoblot of sarkosyl solubility fractionation of brain stem samples from a non-ALS control, ALS-FUS^P525L^ control patient, and ION363-treated ALS-FUS^P525L^ patient. Sol = soluble (in hypotonic buffer), SS = sarkosyl soluble (in 1% sarkosyl and high salt), and SI = sarkosyl insoluble fractions. (**c**) Representative immunoblot of brainstem tissue from a non-ALS control, ALS-FUS^P525L^ control patient, and ION363-treated ALS-FUS^P525L^ patient probed with antibodies against RBPs. Source data

Immunohistochemical staining of the spinal cord and brain sections with an antibody specific to the oligonucleotide backbone of ION363 revealed diffuse predominantly cytoplasmic puncta within neurons (Fig. 5b and Extended Data Fig. 7a). This pattern suggests broad distribution of ION363 throughout the patient’s central nervous system (CNS), and that, at least 2 months after the last therapeutic infusion, the ASO perdures in the treated brain and spinal cord.

Western blot analysis of brainstem tissue from a treated and an untreated ALS-FUS^P525L^ patient compared to normal control tissue (Fig. 5c and Extended Data Fig. 7b) showed a considerable reduction of both total and P525L mutant FUS protein to nearly undetectable levels, showing that, as in the mouse, ION363 can effectively silence FUS in humans. In addition, as we observed in the MN-P517L/Δ14 mutant mice, this ION363-induced downregulation of FUS also resulted in a decrease in the levels of sarkosyl-insoluble FUS and other RBPs, including TDP43, hnRNP A1, hnRNP U and UPF1 (Fig. 5d), but no change in the total protein levels of these RBPs, suggesting their redistribution toward a more soluble state (Extended Data Fig. 7c).

**Extended Data Fig. 8:**
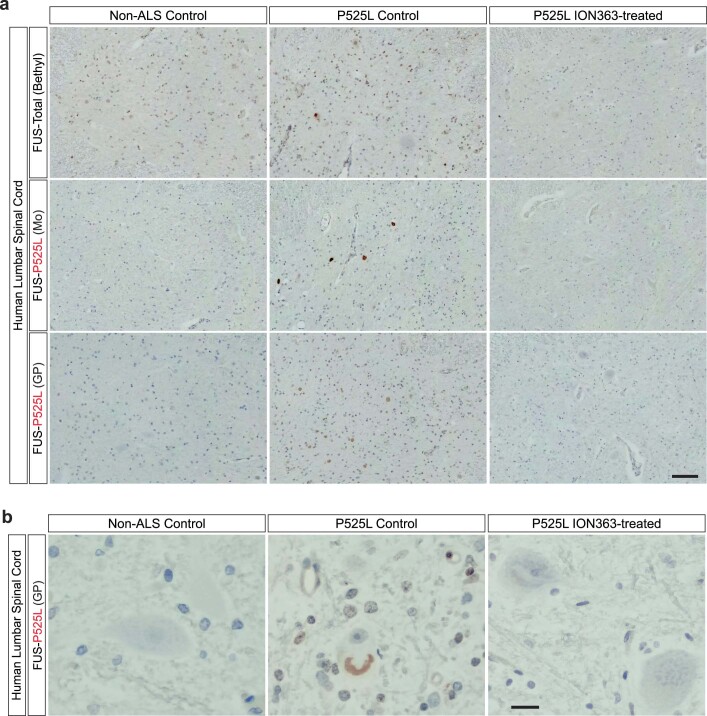
The effect of ION363 on FUS expression and pathology in human lumbar spinal cord. (**a**) Low-power immunohistochemical images of FFPE sections of lumbar spinal cord from a non-ALS control (left), ALS-FUS^P525L^ control patient (middle), and ION363-treated ALS-FUS^P525L^ patient (right) stained with an antibody against total FUS (FUS-Bethyl[400–450], top row), P525L-specific mouse monoclonal antibody reactive to FUS aggregates (middle row), and P525L-specific guinea pig antiserum (bottom row). Scale bar=100 µm. **(b)** Immunohistochemical staining of FFPE sections from lumbar spinal cord of a non-ALS control, ALS-FUS^P525L^ control patient, and ION363-treated ALS-FUS^P525L^ patient with P525L-specific guinea pig antiserum. Scale bar=20 µm.

**Extended Data Fig. 9:**
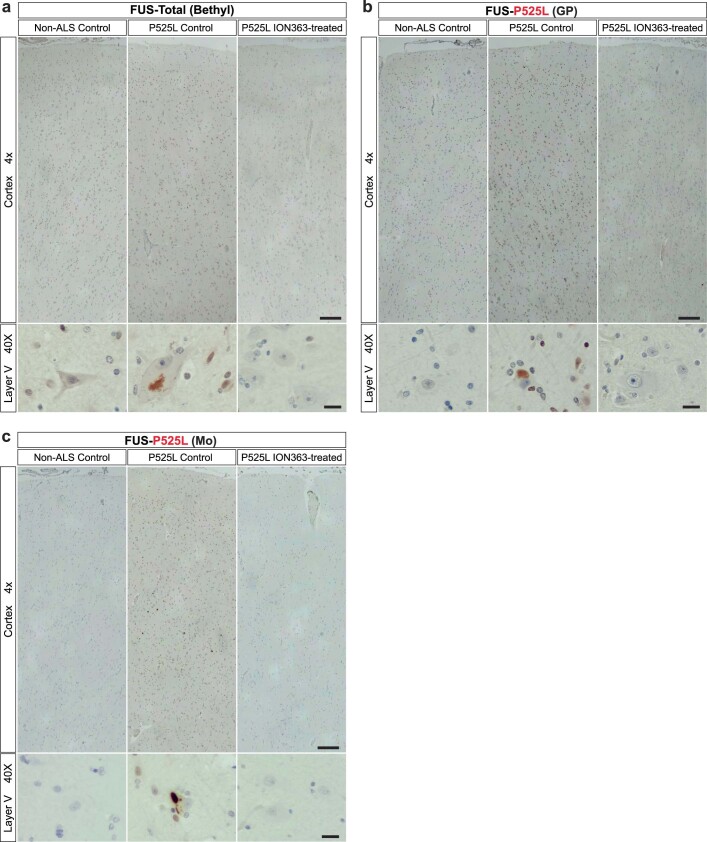
The effect of ION363 on FUS expression and pathology in human motor cortex. (**a**) Immunohistochemical staining of FFPE sections of motor cortex (BA4) from a non-ALS control (left), ALS-FUS^P525L^ control patient (middle), and ION363-treated ALS-FUS^P525L^ patient (right) with an antibody against total FUS (FUS-Bethyl[400–450]). Scale bar=200 µm at 4x and 20 µm at 40x. (**b**) Immunohistochemical staining of FFPE sections of motor cortex (BA4) from a non-ALS control (left), ALS-FUS^P525L^ control patient (middle), and ION363-treated ALS-FUS^P525L^ patient (right) with P525L-specific guinea pig antiserum. Scale bar=200 µm at 4x and 20 µm at 40x. (**c**) Immunohistochemical staining of FFPE sections of motor cortex (BA4) from a non-ALS control (left), ALS-FUS^P525L^ control patient (middle), and ION363-treated ALS-FUS^P525L^ patient (right) with P525L-specific mouse monoclonal antibody. Scale bar=200 µm at 4x and 20 µm at 40x.

Based on the treatment of a single patient, it is not possible to determine if the silencing of FUS had any effect on the course of disease in this case. However, to look for evidence in support of clinical benefit, we asked if the downregulation of FUS had any effect on FUS pathology in the ASO-treated brain. Immunohistochemical staining with a commercial antibody (‘FUS-Total’) showed predominantly nuclear localization of FUS in the spinal cord of the normal control and, in the untreated P525L tissue, numerous abnormal cytoplasmic and intranuclear aggregates within neurons and other cell types—the pathological hallmark of ALS-FUS. In striking contrast, we observed no nuclear FUS staining in the ION363-treated P525L spinal cord and very sparse FUS-positive aggregates in the remaining MNs (Fig. 5e and Extended Data Fig. 8a,b). In the motor cortex of the P525L control brain, FUS localization was also predominantly nuclear, and widespread pathological inclusions were especially prominent in layer V cells. However, in the motor cortex of the ION363-treated P525L case, immunohistochemical staining revealed no nuclear FUS and only scant aggregates within layer V (Extended Data Fig. 9a). Using a P525L-specific polyclonal antibody, a similar pattern was observed in both brain and spinal cord (Extended Data Figs. 8a,b and 9b). Finally, a P525L-specific mouse monoclonal antibody showed a preferential affinity for mutant FUS-positive aggregates in the anterior horn of the untreated P525L spinal cord. However, consistent with previous observations, no abnormal FUS staining was observed in the spinal cord of the ION363-treated case (Fig. 5e and Extended Data Figs. 8a and 9c).

Overall, these data show that ION363 effectively suppresses FUS expression throughout the CNS, reverses biochemical insolubility of RBPs associated with mutant FUS toxicity and reduces the burden of FUS pathological aggregation in surviving, vulnerable neurons in the brain and spinal cord. Together with pre-clinical data from the FUS-ALS knock-in mouse models of disease, this first neuropathological study of a single patient with ALS-FUS treated with ION363 provides preliminary evidence in support of FUS silencing as a promising therapeutic strategy to delay or prevent disease onset in pre-symptomatic carriers of ALS-associated FUS mutations and to slow disease progression in patients with ALS-FUS^32^.

## Discussion

Since the initial discovery of ALS-associated mutations in *FUS*, attention has focused on the possibility that loss of one or more of the many functions of this essential regulatory protein in transcriptional regulation, pre-mRNA splicing, RNA processing and RNA localization might lead to selective neurodegeneration in ALS and FTD. Previous work in our group and others has argued that FUS-mediated toxicity is a not a consequence of FUS loss of function but, rather, a toxic gain of function related the disease-causing mutations. Here, using a series of knock-in mutant mice, we provide genetic evidence that the effect of disease-causing mutations on FUS activity are complex, leading to both loss and gain of function. We show that FUS-P517L (equivalent to human P525L) and the truncation mutation Δ14—both highly pathogenic and associated with aggressive, juvenile-onset ALS—lead to a partial loss of function, each able to rescue the perinatal lethality of FUS deficiency only partially. Despite this loss of function, genetic complementation experiments show that the toxicity of the mutant FUS protein is not primarily a consequence of FUS deficiency but, rather, of a dose-dependent gain of function that leads to enhanced perinatal lethality unrelated to MN degeneration. Although gain-of-function versus loss-of-function mechanisms cannot be fully distinguished here, our data show that FUS toxicity in vivo does not correlate with functional activity. FUS-P517L, although better able to rescue the FUS knockout phenotype, is more toxic than the less functional Δ14 mutant FUS protein. Furthermore, our data find no correlation in vivo between the degree of cytoplasmic mislocalization and toxicity of mutant FUS. The truncated Δ14 protein, which completely lacks the PY-NLS domain, is present in the cytoplasm to a greater degree than FUS-P517L but is less toxic, arguing against mislocalization as a primary determinant of FUS toxicity.

In heterozygous P517L and Δ14 mice, we observe selective, age-dependent MN degeneration involving neuronal subpopulations, including FF, large-diameter alpha-MNs that are preferentially vulnerable in patients with ALS and in other mouse models of ALS. Using the conditional Δ14 (c14) allele, we were able to avoid the developmental toxicity observed in the homozygous P517L and Δ14 mutants and the P517L/Δ14 complex heterozygotes. Selective activation of the c14 mutant allele using ChAT-Cre in the P517L and Δ14 heterozygotes generated mutants in which post-mitotic MNs express two copies of mutant FUS. Analysis of these conditional mutants reveals the dose dependence of mutant FUS-mediated neurodegeneration through the accelerated loss of spinal MNs that nevertheless remains self-limited and selective to the same vulnerable subpopulation of MNs involved in the P517L and Δ14 heterozygous mutants. Moreover, our analysis of the c14/c14 homozygous, ChAT-Cre animals in which only MNs express mutant Δ14 FUS was remarkably similar in phenotype to the Δ14/c14, ChAT-Cre mutants in which a single copy of the Δ14 FUS allele is expressed broadly in the animal, in terms of the onset, time course and extent of denervation and MN loss but also with regard to the astrocyte and microglial proliferation that precedes MN degeneration. These data provide compelling genetic evidence that the onset and progression of MN loss in this mouse model of FUS-ALS is a cell-autonomous process and that, to the extent that other cells (for example, astrocytes and microglia) contribute to neurodegeneration, this occurs independent of the expression of mutant FUS, with no evidence of a cell-non-autonomous mechanism.

The mechanism by which mutations in *FUS*, *TARDBP* (TDP-43) and other genes encoding RBPs cause neuronal degeneration in ALS remains unclear, but a compelling model of disease argues that toxicity involves the formation of abnormal assemblies or condensates of protein and RNA that arise as a consequence of a natural process of LLPS that is critical for the normal, reversible functions of these regulatory proteins. The phase transition behavior of FUS and other ALS-associated proteins depends, in large part, on intrinsically disordered, prion-like domains that govern the interaction of FUS with itself or other partners. According to this model, disease-associated mutations in FUS alter the properties of the protein, leading to aberrant phase transitions—and both loss and gain of function—and increasing the proportion of total protein in condensates, leading to further consolidation and growth of fibrillar structures that are the pathological hallmark of ALS. Although it is not possible to assess the phase or material properties of FUS in vivo, we are able to determine the solubility of FUS and its interactors in the brain and spinal cord of the FUS knock-in mutants, which might reflect the phase state and/or material properties of these proteins. We provide evidence that mutant FUS causes a dose-dependent increase in insoluble FUS and related proteins, along with aberrant splicing events related to the deficiency of those RBPs. Although the observed levels of insoluble FUS/RBPs constitute a very small proportion of total FUS/RBPs, we hypothesize that the insoluble fraction corresponds to the formation of dense condensates that underlie the FUS-dependent toxicity that we observe in this in vivo model of FUS-ALS. Supporting the disease relevance of this finding, the insolubility of FUS and other interacting proteins that we observe in the FUS mice is also evident in the brain and spinal cord of a FUS^P525L^ ALS patient, in which we find biochemical insolubility similar to that which we reported in the CNS of patients with ALS/FTD with the C9orf72 expansion as well as sporadic cases that we found to be like C9. Together, these data suggest an intriguing model of disease in which the insolubility of ALS-associated RBPs are interdependent—consistent with a biophysical model of heterotypic phase separation—where the insolubility/phase transition of a single disordered protein drives the insolubility/condensation of related proteins, leading to widespread loss of function and dysregulation of critical pathways, leading to neurodegeneration.

The saturation concentration (C_sat_) of proteins that undergo LLPS is the critical concentration required for phase separation. ALS-associated mutations in FUS lower the C_sat_ of the protein in vitro, leading to aberrant phase transition and, likely, to associated toxicity in vivo. Our demonstration of dose-dependent toxicity of mutant FUS in the mouse is consistent with a disease model that involves aberrant phase transition and the formation of abnormal assemblies that are toxic to MNs. This model suggests a therapeutic approach to ALS-FUS by which reduction of FUS levels below the C_sat_ could alter the dynamics of LLPS and aggregation and, thereby, delay or prevent disease onset or slow (or halt) disease progression once it has begun. Using an ASO that targets the FUS transcript in a non-allele-specific manner (ION363), we show that a single ICV injection of the therapeutic into the newborn P517L/c14, ChAT-Cre mutant results in marked (50–80%) reduction in total FUS (WT and mutant) and prevents MN degeneration for as long as the ASO continues to silence FUS expression in the CNS. Indeed, 4 months after injection of the ASO, when we consistently see loss of 10–15% of all L4/5 lumbar MNs, there is virtually no evidence of neurodegeneration or the gliosis and muscle denervation that precedes it. At 6 months, when ASO levels are no longer detectable and FUS levels have returned to normal, we begin to see astrogliosis and microgliosis along with TA denervation but still no evidence of MN loss.

With support of these pre-clinical data, we were able to introduce ION363 into the clinic to treat a patient with ALS with a FUS^P525L^ mutation under an FDA-approved, individual-patient, expanded access (‘compassionate use’) protocol. The patient received repeated IT infusions of the FUS-ASO over a 10-month period, without any related, serious adverse events, but unfortunately died of complications of ALS. Analysis of postmortem brain and spinal cord revealed a profound reduction in both WT and mutant FUS protein to levels that were barely detectable immunohistochemically or by western blot analysis. Strikingly, the FUS-positive neuronal cytoplasmic inclusions that are a hallmark of ALS-FUS and prominent in the surviving MNs of the untreated FUS^P525L^ tissue that we analyzed were rarely observed in the ASO-treated case. These data show the efficacy of IT delivery of ION363 in lowering the levels of the pathogenic FUS protein and the tolerance of our patient to prolonged reduction in FUS levels in the CNS. Moreover, this first-in-human study of this antisense therapeutic shows that FUS silencing leads to a reduction in the burden of FUS pathology in the ALS-FUS brain, altering the equilibrium between formation and clearance of abnormal FUS aggregates to restore balance in vulnerable neurons. Although this might have been too little and too late to change the clinical outcome in our patient, these neuropathological findings, together with the mouse data showing a profound effect of ION363 on the FUS MN phenotype, underlie the therapeutic potential of this antisense therapeutic. An ongoing, pivotal, phase 3 clinical study of ION363 (NCT04768972) will determine whether this antisense therapeutic provides any clinical benefit of slowing disease progression in symptomatic patients with ALS-FUS. Future studies will determine whether FUS silencing with this ASO delays disease onset in pre-symptomatic carriers of ALS-associated FUS mutations as it did in the mouse.

## Methods

### Human participants, tissue samples and ION363 drug

Written informed consent for treatment of the patient with ION363 was obtained under an investigational protocol approved by the FDA and the institutional review board of Columbia University Irving Medical Center. This consent included the use of clinical data and de-identified demographic information for research purposes, as contained in this publication. All postmortem data were generated from tissue samples collected and banked at the New York Brain Bank of Columbia University with consent obtained from the patient’s next of kin, according to New York State law and the guidelines of the Department of Pathology of Columbia University and New York Presbyterian Hospital. In the State of New York, research involving autopsy material does not meet the regulatory definition of ‘human subject research’ and is not subject to institutional review board oversight.

### ION363 (Jacifusen) drug

ION363 is a sodium salt of an oligonucleotide with a chemically modified backbone to improve its stability^33^. Its full chemical name is O2′-(2-methoxyethyl)-P-thio-guanylyl-(3′->5′)-O2′-(2-methoxyethyl)-5-methyl-cytidylyl-(3′->5′)-O2′-(2-methoxyethyl)-adenylyl-(3′->5′)-O2′-(2-methoxyethyl)-adenylyl-(3′->5′)-O2′-(2-methoxyethyl)-5-methyl-uridylyl-(3′->5′)-O2′-(2-deoxy)-P-Thio-guanylyl-(3′->5′)-O2′-(2-deoxy)-P-Thio-thymidinyl-(3′->5′)-O2′-(2-deoxy)-5-Methyl-P-Thio-cytidylyl-(3′->5′)-O2′-(2-deoxy)-P-Thio-adenylyl-(3′->5′)-O2′-(2-deoxy)-5-Methyl-P-Thio-cytidylyl-(3′->5′)-O2′-(2-deoxy)-5-Methyl-P-Thio-cytidylyl-(3′->5′)-O2′-(2-deoxy)-P-Thio-thymidinyl-(3′->5′)-O2′-(2-deoxy)-P-Thio-thymidinyl-(3′->5′)-O2′-(2-deoxy)-P-Thio-thymidinyl-(3′->5′)-O2′-(2-deoxy)-5-Methyl-P-Thio-cytidylyl-(3′->5′)-O2′-(2-methoxyethyl)-adenylyl-(3′->5′)-O2′-(2-methoxyethyl)-5-methyl-uridylyl-(3′->5′)-O2′-(2-methoxyethyl)-P-Thio-adenylyl-(3′->5′)-O2′-(2-methoxyethyl)-5-methyl-P-Thio-cytidylyl-(3′->5′)-O2′-(2-methoxyethyl)-5-methyl-cytidine sodium salt. The sequence of ION363 corresponds to the 5′-GCAATGTCACCTTTCATACC-3′ sequence within the sixth intron of FUS gene. The molecular weight of ION363 is 7,477.4 Da.

ION363 was manufactured by ChemGenes Corporation according to Good Manufacturing Practice standards. For the animal experiments, drug powder was reconstituted in sterile PBS to a total concentration of 4 mg ml^−1^. For the administration to the patient, ION363 was reconstituted in a USP compendial grade artificial cerebrospinal fluid to a total concentration of 11 mg ml^−1^. The appropriate drug volume for 20-mg, 40-mg and 60-mg doses was subsequently sterilized through a 0.2-µm filter into a sterile pyrogen-free syringe.

### Mouse lines and procedures

All experiments involving live animals were approved by the Institutional Animal Care and Use Committee at Columbia University Irving Medical Center. C57Bl/6J (000664), ChAT-Cre (006410)^28^, ChAT-CreΔneo (031661), protamine-Cre (003328)^34^ and Pgk1-flpo (011065)^35^ mouse lines were obtained from Jackson Laboratory. All strains were backcrossed to C57Bl/6J background for at least five generations. All animals were housed in a specific pathogen-free facility with ambient temperature of 18–23 °C and 40–60% humidity and a 12-h light/dark cycle.

Mutant FUS knock-in mice were generated by targeting of the endogenous mouse FUS locus by homologous recombination. The targeting construct included a ~6.4 kb of C57BL/6 mouse strain genomic FUS sequence spanning exons 11–15, 3′ untranslated region (UTR) and downstream sequence. FUS genomic sequence was subcloned from BAC RP24-297F14 by gap repair recombine, and its border sequences (inclusive) were GGTCACCTCAAATAGTGAGTTTCATG on the 5′ end and CTTGTAGCTCAATTGGGTTGAAATA on the 3′ end. FRT-site flanked neomycin resistance cassette (NEO) was inserted 50 bp downstream of the end of the 3′ UTR for positive selection of drug-resistant embryonic stem cells. For P517L point mutants, single base pair substitution was introduced in FUS exon 15. In the conditional FUS C14 construct, exon 14 was flanked by *LoxP* sites, and part of mouse exon 15 was replaced with human exon 15 sequence to accurately reproduce the novel human C-terminal Δ14 protein sequence when exon 14 is skipped and exon 15 is read out of frame in the FUS Δ14 mouse. Targeting constructs were electroporated into GFP-expressing LB10 mouse embryonic stem cells derived from a C57BL6/N mouse (GlobalStem, GSC-5003), and recombinants were detected by Southern blot.

FUS^KO^ (EUCOMM allele FUS^tm1d^)^12^ and Δ14 mouse lines were generated by first crossing FUS^FLOX^ (EUCOMM allele FUS^tm1c^) or FUS C14 alleles to protamine-Cre line and subsequently crossing FUS^FLOX^/WT or C14/WT; protamine-Cre-positive males to C57Bl/6J females.

All primer sequences used for genotyping are listed in Supplementary Table 1.

Motor function evaluation was performed using the grip strength test (Bioseb, BIO-GS3).

For ICV injections, newborn animals were first anesthetized on ice, and then 20 μg of ION363 or NTC oligonucleotide in a total volume of 5 μl was injected into the right lateral ventricle (2 mm frontal and 1 mm lateral to bregma) using a Hamilton Neuros syringe. After the injection, animals were placed on a heating pad and monitored until recovery.

To collect fixed tissue samples, the animals were transcardially perfused with PBS-heparin solution followed by 4% paraformaldehyde; brain, spinal cord and muscles (diaphragm, TA and soleus) were dissected.

### PCR and RT–PCR

RNA from brain or spinal cord samples was isolated with TRIzol reagent (Invitrogen); its concentration was measured using NanoDrop 2000 (Thermo Fisher Scientific); and cDNA was generated with SuperScript IV reverse transcriptase (Invitrogen) according to the manufacturer’s protocol. GoTaq G2 Hot Start Master Mix (Promega) and PowerUp SYBR Green Master Mix (Thermo Fisher Scientific) were used to set up PCR or qPCR reactions, respectively. All primers used in this study are listed in Supplementary Table 1.

### Immunofluorescence

Spinal cord samples were sectioned at 70-µm thickness on a Leica VT 1000S vibratome. TA and soleus muscles were sectioned at 35-µm thickness on a Leica CM 3050S cryostat. Diaphragms were stained as whole mount.

Tissue sections were incubated in primary antibodies (Supplementary Table 2) diluted in 5% normal donkey serum in Tris-buffered saline with 0.5% Triton X-100 (TBS-T) overnight at 4 °C. The sections were then washed in TBS-T three times and incubated with the corresponding secondary antibodies (Supplementary Table 3) for 1 h. After three washes in TBS-T, the sections were mounted in aqueous medium (Fluoromount G, Southern Biotech) and imaged using a Leica SP8 confocal microscope. Images were analyzed using LAS X and ImageJ software packages.

### Western blotting

Tissue lysates were mixed with 4× sodium dodecyl sulfate (SDS) sample buffer, incubated at 100 °C for 5 min and run on Novex WedgeWell Tris-Glycine gels (4–20%, 8–16% or 10–20%) and transferred onto nitrocellulose membranes (Thermo Fisher Scientific) using mini-blot modules (Thermo Fisher Scientific). The membranes were then blocked in Pierce Protein-Free T20 (TBS) Blocking Buffer (Thermo Fisher Scientific) and incubated with the corresponding primary antibodies (Supplementary Table 2) overnight at 4 °C. Then, the membranes were washed in TBS with 0.1% Tween 20 three times and incubated with HRP-conjugated secondary antibodies (Supplementary Table 3) for 1 h. After three washes in TBS-Tween 20, the membranes were treated with SuperSignal West Pico or SuperSignal West Femto (Thermo Fisher Scientific) and imaged using an iBright CL1000 chemiluminiscence imaging system (Thermo Fisher Scientific).

### Nucleo-cytoplasmic fractionation

This protocol was adapted from Shiihashi et al.^36^. Frozen tissue samples were weighed and homogenized in 10 μl per 1 mg of tissue of cytoplasmic buffer (10 mM HEPES, 10 mM NaCl, 1 mM KH_2_PO_4_, 5 mM NaHCO_3_, 5 mM EDTA, 1 mM CaCl_2_, 0.5 mM MgCl_2_). After incubating the homogenized tissue on ice for 10 min, 0.5 μl mg^−1^ of 2.5 M sucrose was added and centrifuged at 6,300*g* for 10 min. The supernatant was collected as the cytoplasmic fraction, and the pellet was washed three times with the nuclear buffer (10 mM Tris pH 7.4, 300 mM sucrose, 1 mM EDTA, 0.1% Nonidet-40), homogenized and centrifuged at 1,000*g* for 5 min. The pellet was resuspended in 5 μl per 1 mg of tissue of immunoprecipitation buffer containing 2% SDS as the nuclear fraction.

### Subcellular fractionation with sarkosyl

This protocol was adapted from Conlon et al.^37^. Tissue samples were weighed and, for every milligram of tissue, 18 μl of ice-cold soluble buffer (0.1 M MES pH 7, 1 mM EDTA, 0.5 mM MgSO_4_, 1 M sucrose) containing 50 mM N-ethylmaleimide (NEM), 1 mM NaF, 1 mM Na_3_VO_4_, 1 mM PMSF and Halt protease and phosphatase inhibitor cocktail (Thermo Fisher Scientific) was added. Samples were homogenized by several passages through needles with decreasing diameter (20-gauge, 23-gauge and 27.5-gauge needles). An equivalent volume of the homogenate (700 μl) was collected and centrifuged at 50,000*g* for 20 min at 4 °C. The supernatant was collected as a soluble fraction. The pellet was resuspended in 700 μl of RAB buffer (100 mM MES pH 6.8, 10% sucrose, 2 mM EGTA, 0.5 mM MgSO_4_, 500 mM NaCl, 1 mM MgCl_2_, 10 mM NaH_2_PO_4_, 20 mM Na, 1 mM PMSF, 50 mM NEM) containing 1% of *N*-lauroylsarcosine (sarkosyl) and Halt protease and phosphatase inhibitor cocktail (Thermo Fisher Scientific), vortexed for 1 min at room temperature and incubated at 4 °C overnight with end-over-end rotation. The samples were then centrifuged at 200,000*g* for 30 min at 12 °C, and the supernatant was collected as the sarkosyl-soluble fraction. The pellet was resuspended in 700 μl of RAB buffer and passed 3–5 times through a 27.5-gauge needle to fully disperse the pellet, creating the sarkosyl-insoluble fraction.

### Immunohistochemistry

Five-micron tissue sections were deparaffinized in xylene and rehydrated in ethanol solutions with decreasing concentrations. Antigen retrieval was performed by autoclaving the slides in Tris-EDTA buffer (10 mM Tris pH 9, 1 mM EDTA, 0.05% Tween 20) for 10 min. The slides were then washed in TBS and incubated with primary antibodies (Supplementary Table 2) in TBS solution with 0.01% Triton X-100 and 5% normal goat serum overnight at 4 °C. The next day, the slides were washed three times in TBS and incubated with HRP-conjugated secondary antibodies (Supplementary Table 3). After three washes in TBS, the slides were developed with DAB Substrate Kit (Thermo Fisher Scientific) for 10 min and counterstained with Mayer’s hematoxylin for 2 min. The slides were then immediately rinsed in tap water for 5 min, dehydrated in ethanol solutions with increasing concentrations followed by xylenes and mounted with Organo/Limonene Mount (Millipore Sigma).

### Statistics and reproducibility

Statistical analysis was performed using the GraphPad Prism 9.0.2 software package. All experiments were performed in at least three technical replicates and using at least three animals per group where applicable.

### Reporting Summary

Further information on research design is available in the Nature Research Reporting Summary linked to this article.

## Online content

Any methods, additional references, Nature Research reporting summaries, source data, extended data, supplementary information, acknowledgements, peer review information; details of author contributions and competing interests; and statements of data and code availability are available at 10.1038/s41591-021-01615-z.

## Supplementary information

Reporting Summary

Supplementary Tables Supplementary tables listing oligonucleotides and antibodies used in the study.

## Source data

Source Data Fig. 1 Unprocessed western blots.

Source Data Fig. 2 Unprocessed western blots.

Source Data Fig. 4 Unprocessed western blots.

Source Data Fig. 5 Unprocessed western blots.

Source Data Extended Data Fig. 1 Unprocessed western blots.

Source Data Extended Data Fig. 2 Unprocessed western blots.

Source Data Extended Data Fig. 4 Unprocessed western blots.

Source Data Extended Data Fig. 6 Unprocessed western blots.

Source Data Extended Data Fig. 7 Unprocessed western blots.

## Data Availability

All data generated or analyzed during this study are included in this published article and its Supplementary Information files. All requests for raw and analyzed data and materials should be addressed to the corresponding author and will be reviewed by the intellectual property and privacy offices of Columbia University to verify whether the request is subject to any intellectual property or confidentiality obligations. Patient data might be subject to patient confidentiality. Raw clinical data are stored at Columbia University Irving Medical Center with indefinite appropriate backup. Patient-related data not included in the paper were generated as part of an expanded access treatment protocol and might be subject to patient confidentiality. Any data and materials that can be shared will be released via a material transfer agreement. Source data are provided with this paper.
