# Supplementary material for: Antisense oligonucleotide silencing of FUS expression as a therapeutic approach in amyotrophic lateral sclerosis
Source: Nat Med. 2022 Jan 24;28(1):104–16. doi: 10.1038/s41591-021-01615-z (PMC8799464; doi:10.1038/s41591-021-01615-z)
Supplement: Supplementary file 1 — Reporting Summary [file 41591_2021_1615_MOESM1_ESM.pdf]

Corresponding author(s): Neil A Shneider MD PhD

Last updated by author(s): Nov 1, 2021

Reporting Summary

Nature Portfolio wishes to improve the reproducibility of the work that we publish. This form provides structure for consistency and transparency in reporting. For further information on Nature Portfolio policies, see our Editorial Policies and the Editorial Policy Checklist.

Statistics

For all statistical analyses, confirm that the following items are present in the figure legend, table legend, main text, or Methods section.

n/a	Confirmed	
☐	☒	The exact sample size (*n*) for each experimental group/condition, given as a discrete number and unit of measurement
☐	☒	A statement on whether measurements were taken from distinct samples or whether the same sample was measured repeatedly
☐	☒	The statistical test(s) used AND whether they are one- or two-sided<br>*Only common tests should be described solely by name; describe more complex techniques in the Methods section.*
☐	☒	A description of all covariates tested
☐	☒	A description of any assumptions or corrections, such as tests of normality and adjustment for multiple comparisons
☐	☒	A full description of the statistical parameters including central tendency (e.g. means) or other basic estimates (e.g. regression coefficient) AND variation (e.g. standard deviation) or associated estimates of uncertainty (e.g. confidence intervals)
☐	☒	For null hypothesis testing, the test statistic (e.g. *F*, *t*, *r*) with confidence intervals, effect sizes, degrees of freedom and *P* value noted<br>*Give P values as exact values whenever suitable.*
☒	☐	For Bayesian analysis, information on the choice of priors and Markov chain Monte Carlo settings
☒	☐	For hierarchical and complex designs, identification of the appropriate level for tests and full reporting of outcomes
☒	☐	Estimates of effect sizes (e.g. Cohen's *d*, Pearson's *r*), indicating how they were calculated

Our web collection on statistics for biologists contains articles on many of the points above.

Software and code

Policy information about availability of computer code

Data collection	LAS X (3.7.4) software for confocal imaging using Leica SP8 microscope.
Data analysis	GraphPad Prism 9.0.2 for statistical analysis. LAS X (3.7.4) and ImageJ (1.53) for the analysis of confocal microscopy images. Image Studio (5.2.5) for the analysis of Western blot images.

For manuscripts utilizing custom algorithms or software that are central to the research but not yet described in published literature, software must be made available to editors and reviewers. We strongly encourage code deposition in a community repository (e.g. GitHub). See the Nature Portfolio guidelines for submitting code & software for further information.

Data

Policy information about availability of data

All manuscripts must include a data availability statement. This statement should provide the following information, where applicable:

- Accession codes, unique identifiers, or web links for publicly available datasets
- A description of any restrictions on data availability
- For clinical datasets or third party data, please ensure that the statement adheres to our policy

All data generated or analyzed during this study are included in this published article and its supplementary information files. All requests for raw and analyzed data and materials should be addressed to the corresponding author and will be reviewed by the intellectual property and privacy offices of Columbia University to verify whether the request is subject to any intellectual property or confidentiality obligations. Patient data may be subject to patient confidentiality. Raw clinical data are stored at Columbia University Irving Medical Center with indefinite appropriate backup. Patient-related data not included in the paper were generated as part of an expanded access treatment protocol and might be subject to patient confidentiality. Any data and materials that can be shared will be released via a material transfer agreement.

Field-specific reporting

Please select the one below that is the best fit for your research. If you are not sure, read the appropriate sections before making your selection.

☒ Life sciences ☐ Behavioural & social sciences ☐ Ecological, evolutionary & environmental sciences

For a reference copy of the document with all sections, see nature.com/documents/nr-reporting-summary-flat.pdf

Life sciences study design

All studies must disclose on these points even when the disclosure is negative.

Sample size	No advanced sample size calculation was performed. All mice were continuously generated and included in the experiments required by the project. At minimum, 3 animals per group were used in all experiments.
Data exclusions	No data were excluded from analysis.
Replication	All experiments were performed in at least 3 technical replicates and using at least 3 animals per group where applicable. All replication attempts were successful.
Randomization	Laboratory animals were allocated into groups based on their genotype in a non-random way to specifically avoid a littermate and cage bias and to ensure the inclusion of both sexes into each group.
Blinding	All technical personnel collecting data were blinded. The investigators performed the group allocation and therefore could not be blinded.

Reporting for specific materials, systems and methods

We require information from authors about some types of materials, experimental systems and methods used in many studies. Here, indicate whether each material, system or method listed is relevant to your study. If you are not sure if a list item applies to your research, read the appropriate section before selecting a response.

Materials & experimental systems

n/a	Involved in the study	
☐	☒	Antibodies
☒	☐	Eukaryotic cell lines
☒	☐	Palaeontology and archaeology
☐	☒	Animals and other organisms
☐	☒	Human research participants
☒	☐	Clinical data
☒	☐	Dual use research of concern

Methods

n/a	Involved in the study	
☒	☐	ChIP-seq
☒	☐	Flow cytometry
☒	☐	MRI-based neuroimaging

Antibodies

Antibodies used

All information about the antibodies used in this study, including the manufacturer, species, catalog number, and working dilution for the corresponding application is provided in Supplementary Tables 2 and 3.

Validation

All antibodies used in this study have been previously validated by the manufacturer (listed below) and used in accordance with the manufacturer's recommendation for a giving application. Antibodies were also independently validated in the laboratory by titration on positive and negative controls to optimize their working conditions.

Anti-Synaptopysin (Thermo Fisher Scientific Cat# 180130, RRID:AB_10836766)
Discontinued by the manufacturer in 2018.

Anti-Neurofilament H & M, phosphorylated (Millipore Cat# MAB1592, RRID:AB_94275)
https://www.emdmillipore.com/US/en/product/Anti-Neurofilament-NF-H-Antibody-phosphorylated-Antibody-clone-NP1,MM_NF-MAB1592-C

Anti-Choline Acetyltransferase (Millipore Cat# AB144P, RRID:AB_2079751)
https://www.emdmillipore.com/US/en/product/Anti-Choline-Acetyltransferase-Antibody,MM_NF-AB144P

Anti-Iba1 (FUJIFILM Wako Shibayagi Cat# 019-19741, RRID:AB_839504)
https://labchem-wako.fujifilm.com/us/category/01213.html?gclid=EAIaIQobChMIuNXjkd_H8wIVNwaICR0awQgSEAAYASAAEgIVr_D_BwE

Anti-GFAP (Agilent Cat# Z0334, RRID:AB_10013382)

https://www.agilent.com/en/product/immunohistochemistry/antibodies-controls/primary-antibodies/glial-fibrillary-acidic-protein-(concentrate)-76683

Anti-FUS/TLS (Proteintech Cat# 11570-1-AP, RRID:AB_2247082)
https://www.ptglab.com/Products/FUS-Antibody-11570-1-AP.htm

Anti-FUS/TLS (Abcam Cat# ab84078, RRID:AB_2105201)
https://www.abcam.com/tlsfus-antibody-ab84078.html

Anti-FUS/TLS, 4H11 (Santa Cruz Biotechnology Cat# sc-47711, RRID:AB_2105208)
https://www.scbt.com/p/fus-tls-antibody-4h11

Anti-FUS (Bethyl Cat# A300-302A, RRID:AB_309445)
https://www.bethyl.com/product/A300-302A

Anti-FUS (Bethyl Cat# A300-293A, RRID:AB_263409)
https://www.bethyl.com/product/A300-293A

Anti-Transportin 1 (Novus Cat# NB600-1397, RRID:AB_792394)
https://www.novusbio.com/products/transportin-1-antibody-d45_nb600-1397

Anti-UPF1, D15G6 (Cell Signaling Technology Cat# 12040, RRID:AB_2797806)
https://www.cellsignal.com/products/primary-antibodies/upf1-d15g6-rabbit-mab/12040

Anti-Caprin1 (Proteintech Cat# 15112-1-AP, RRID:AB_2070016)
https://www.ptglab.com/Products/CAPRIN1-Antibody-15112-1-AP.htm

Anti-TDP-43 (Proteintech Cat# 10782-2-AP, RRID:AB_615042)
https://www.ptglab.com/Products/TARDBP-Antibody-10782-2-AP.htm

Anti-hnRNP U (Bethyl Cat# A300-689A, RRID:AB_530292)
https://www.bethyl.com/product/A300-689A

Anti-hnRNP H (Bethyl Cat# A300-511A, RRID:AB_203269)
https://www.bethyl.com/product/A300-511A

Anti-hnRNP A1, 4B10 (Millipore Cat# 05-1521, RRID:AB_10561756)
https://www.emdmillipore.com/US/en/product/Anti-hnRNP-A1-Antibody-clone-4B10,MM_NF-05-1521

Anti-hnRNP K, F45 P9 C7 (Thermo Fisher Scientific Cat# MA1-087, RRID:AB_2120378)
https://www.thermofisher.com/antibody/product/hnRNP-K-Antibody-clone-F45-P9-C7-Monoclonal/MA1-087

Anti-GAPDH (Millipore Cat# MAB374, RRID:AB_2107445)
https://www.emdmillipore.com/US/en/product/Anti-Glyceraldehyde-3-Phosphate-Dehydrogenase-Antibody-clone-6C5,MM_NF-MAB374

Anti-Histone H3 (Abcam Cat# ab1791, RRID:AB_302613)
https://www.abcam.com/histone-h3-antibody-nuclear-marker-and-chip-grade-ab1791.html

Allele-specific anti-FUS antibodies generated for this study (guinea pig P517/P525L and Δ14 antisera and mouse P517/P525L hybridoma) were validated using mouse tissues positive or negative for the studied allele.

Anti-ASO antibody (Butler, M., Stecker, K. & Bennett, C.F., 1997) was provided by Ionis Pharmaceutical and was validated in the laboratory using positive and negative control tissues.

Animals and other organisms

Policy information about studies involving animals; ARRIVE guidelines recommended for reporting animal research

Laboratory animals	FUS knock-in animals were generated for this study as described.<br>C57Bl/6J (#000664), ChAT-Cre (#006410), ChAT-Creneo (#031661), Protamine-Cre (#003328), and Pgk1-flpo (#011065) mouse lines were obtained from Jackson Laboratory (JAX, Bar Harbor, ME).<br>All strains were backrossed to C57Bl/6J background for at least 5 generations.<br>All animals were housed in a specific pathogen free (SPF) facility with ambient temperature 18-23°C and 40-60% humidity and 12-hour light/ 12-hourdark cycle.
Wild animals	No wild animals were used in this study.
Field-collected samples	No field-collected samples were used in this study.
Ethics oversight	All experiments involving life animals were approved by the Institutional Animal Care and Use Committee at Columbia University Irving Medical Center.

Note that full information on the approval of the study protocol must also be provided in the manuscript.

Human research participants

Policy information about studies involving human research participants

Population characteristics	A single 25-year-old female patient with ALS associated with a pathogenic FUS-P525L mutation. Patient with a family history of ALS. Spinal-onset, rapidly progressive disease with bulbar dysfunction and ventilatory insufficiency.
Recruitment	The patient was enrolled as a subject in the ALS Families Study at Eleanor and Lou Gehrig ALS Center at Columbia University Irving Medical Center. This is a longitudinal study of pre-symptomatic carriers of ALS gene mutations. Upon symptomatic conversion, and individual expanded access IND was submitted to the FDA requesting approval to treat the patient with the experimental antisense therapeutic ION363 targeting FUS expression.
Ethics oversight	Treatment of the single human subject with ION363 was approved by the Food and Drug Administration under an individual patient expanded access IND (IND #144179). The protocol was approved by the Institutional Review Board of Columbia University Irving Medical Center, which provided scientific, regulatory and ethical oversight of this treatment protocol.

Note that full information on the approval of the study protocol must also be provided in the manuscript.
