# Supplementary material for: Antisense oligonucleotide silencing of FUS expression as a therapeutic approach in amyotrophic lateral sclerosis
Source: Nat Med. 2022 Jan 24;28(1):104–16. doi: 10.1038/s41591-021-01615-z (PMC8799464; doi:10.1038/s41591-021-01615-z)
Supplement: Supplementary file 3 — Unprocessed western blots. [file 41591_2021_1615_MOESM3_ESM.pdf]

Uncropped images of Western blots.

Figure 1g.

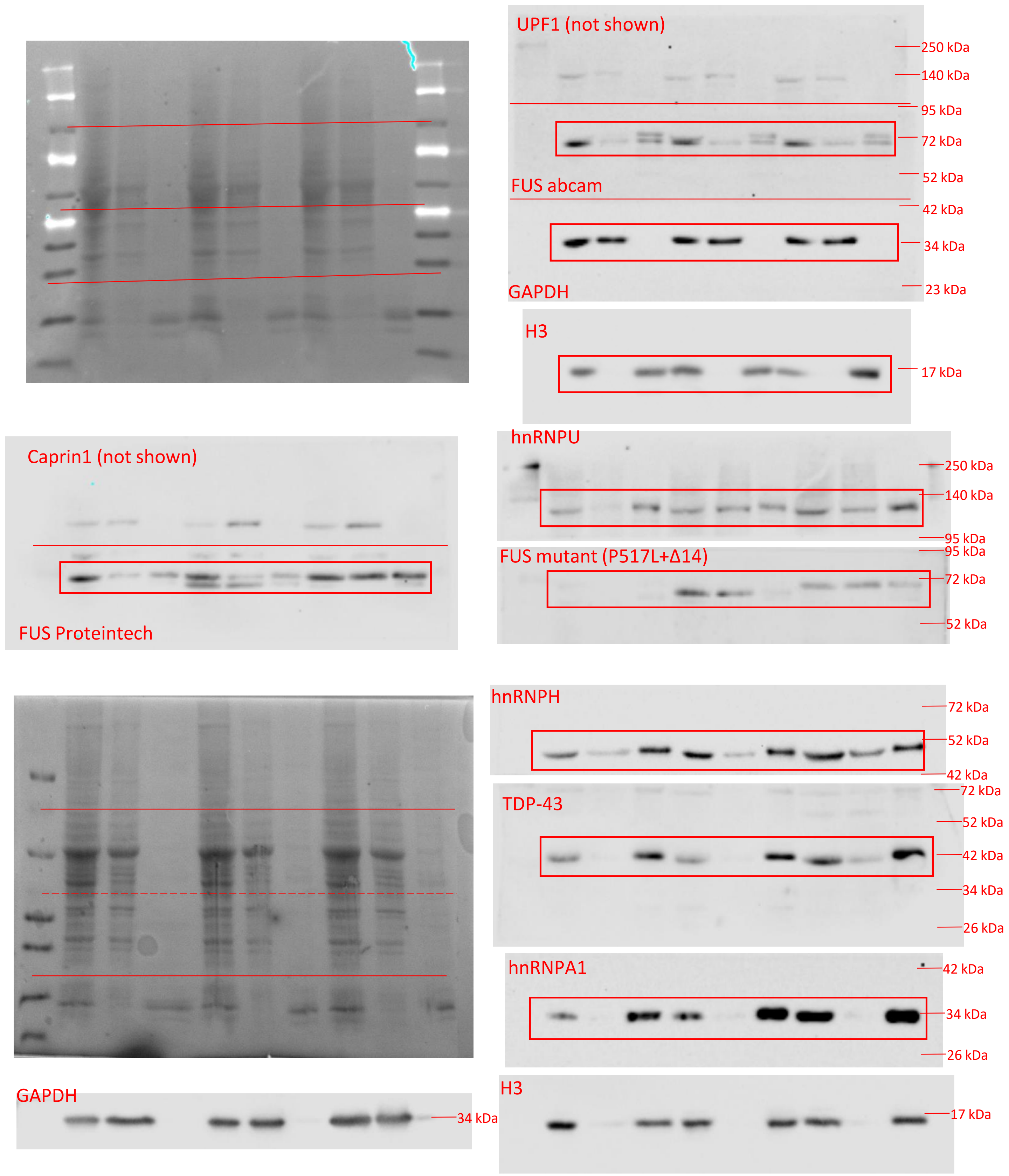

Ponceau and uncropped WB images used for figure 1g. Two membranes with identical sample loading were used to generate the display image. Total, cytoplasmic, and nuclear fractions were loaded side by side for the following genotypes: WT (lanes 1-3), Δ14/WT (lanes 4-6) and P517L/WT (lanes 7-9). The membranes were cut and probed with antibodies according to indications in the figure . Dashed line indicates cutting the membrane after the first probing with TDP43 antibodies for subsequent re-probing with hnRNPH, hnRNPA1, and GAPDH antibodies.
