# Supplementary material for: Antisense oligonucleotide silencing of FUS expression as a therapeutic approach in amyotrophic lateral sclerosis
Source: Nat Med. 2022 Jan 24;28(1):104–16. doi: 10.1038/s41591-021-01615-z (PMC8799464; doi:10.1038/s41591-021-01615-z)

Uncropped images of Western blots.

Figure 2c.

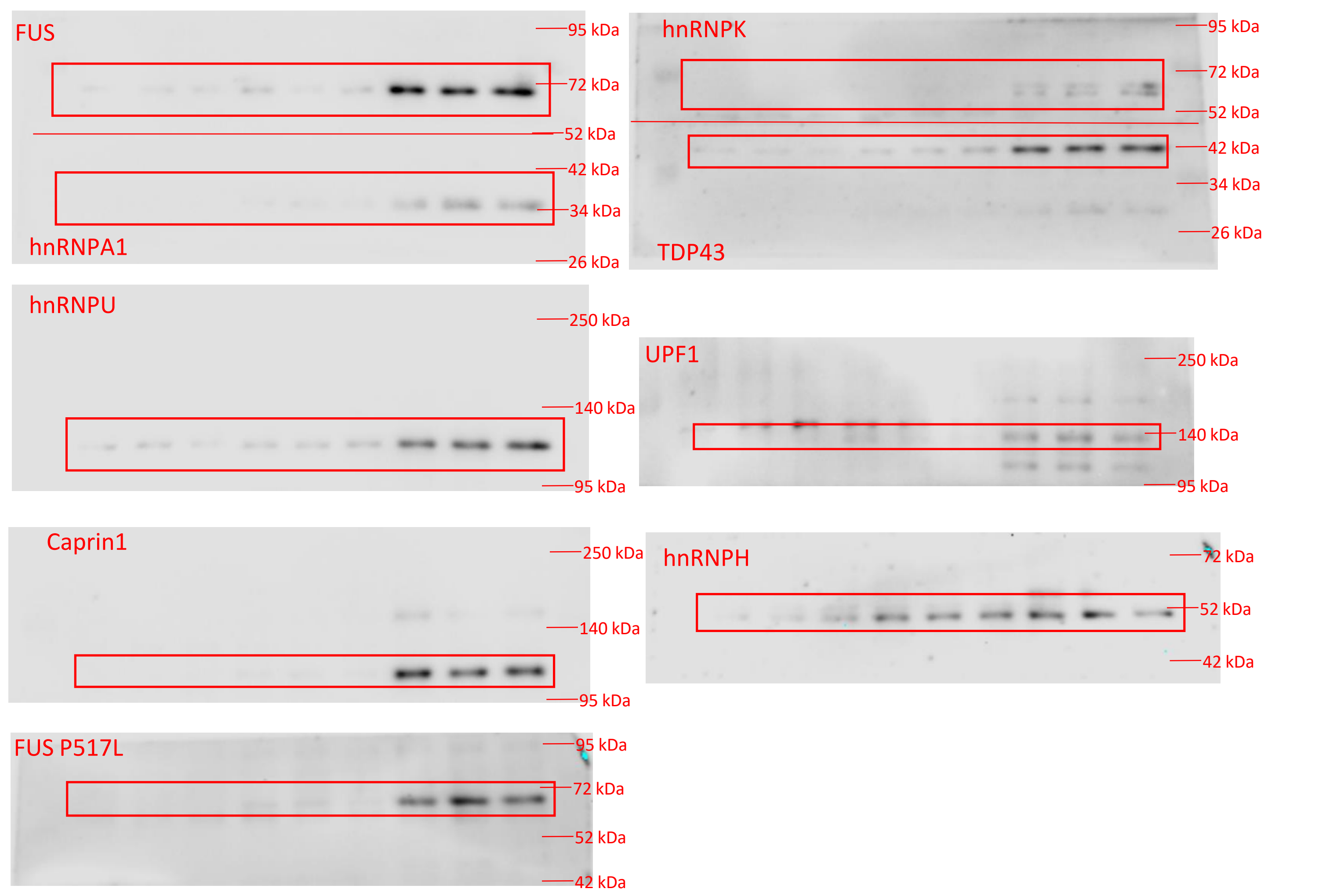

Uncropped WB images used for figure 2c. The intensity levels were adjusted to highlight low-intensity bands. Equal volumes of sarkosyl insoluble fractions from the brains of P0 animals were loaded; each lane represents a different animal. Lanes 1-3 – WT, lanes 4-6 – P517L/WT, and lanes 7-9 – P517L/P517.

Figure 2d.

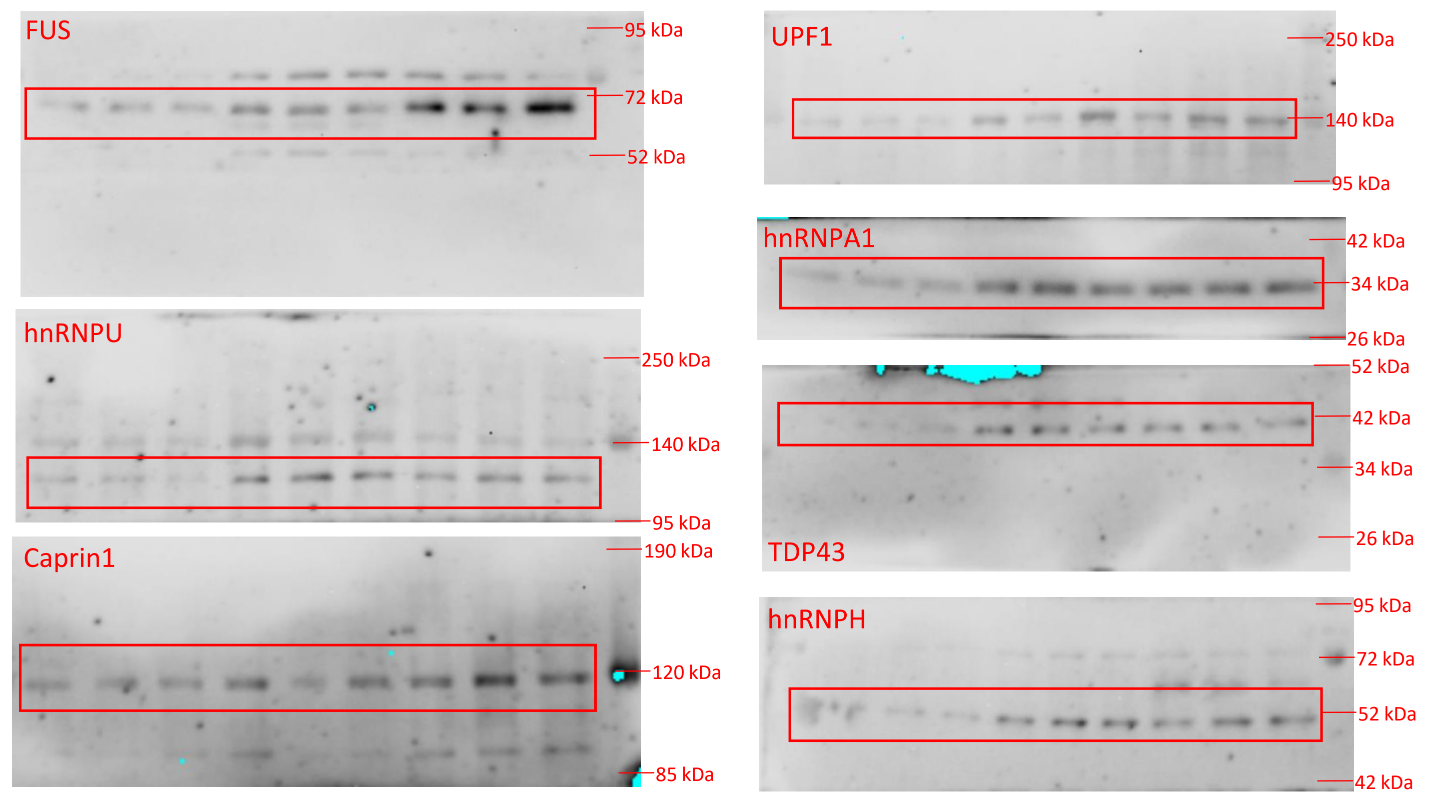

Figure 2e.

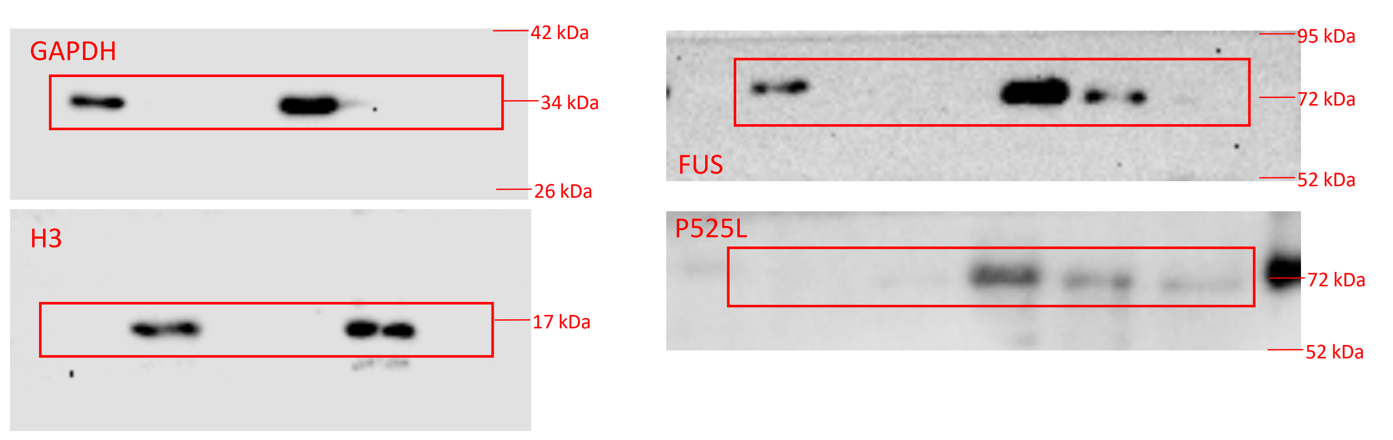

Figure 2f.

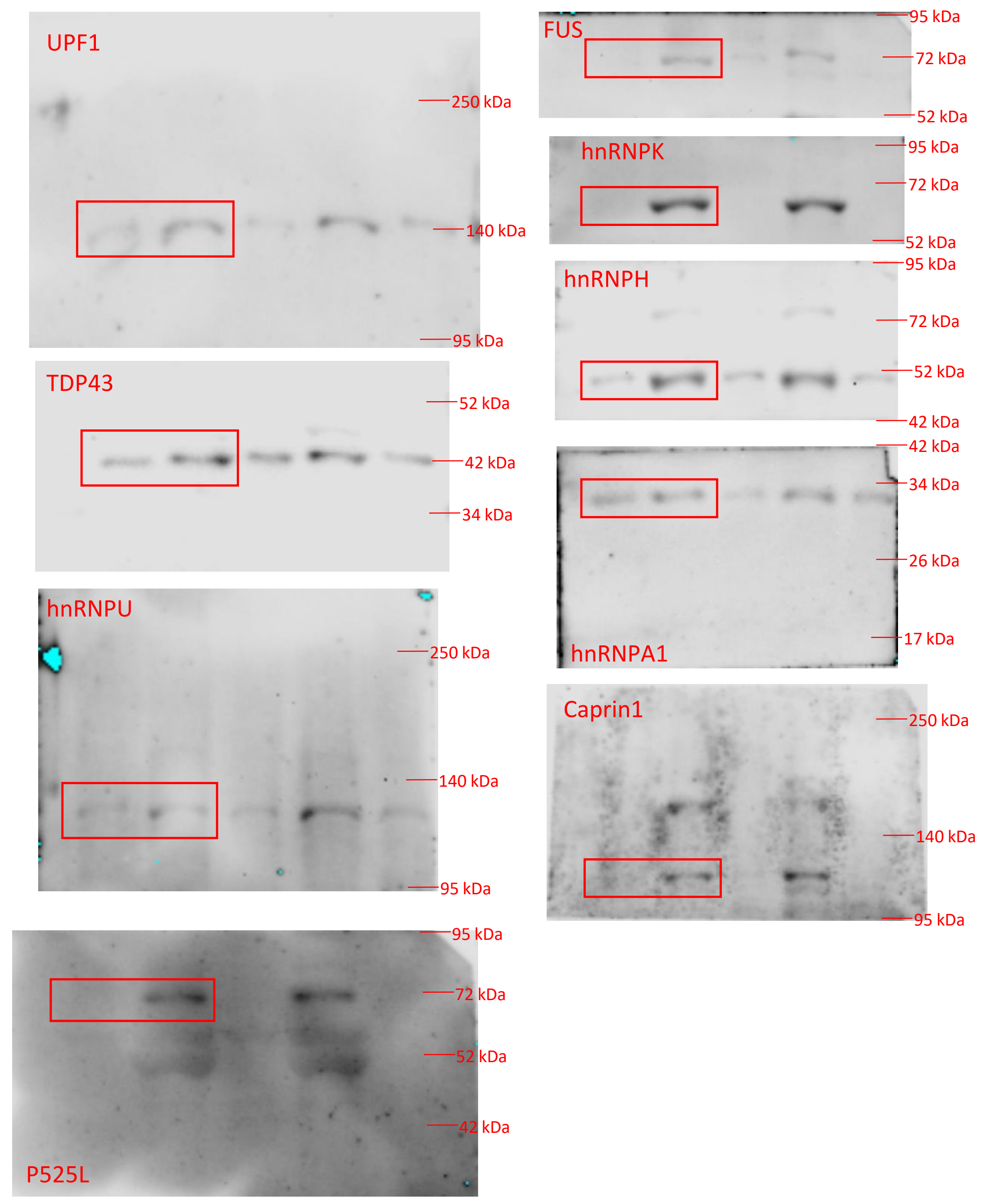

Supplement: Supplementary file 4 — Unprocessed western blots. [file 41591_2021_1615_MOESM4_ESM.pdf]
