# Supplementary figures and images for: Antisense oligonucleotide silencing of FUS expression as a therapeutic approach in amyotrophic lateral sclerosis
Source: Nat Med. 2022 Jan 24;28(1):104–16. doi: 10.1038/s41591-021-01615-z (PMC8799464; doi:10.1038/s41591-021-01615-z)

Uncropped images of Western blots.

Figure 4a. Ponceau stain and uncropped Western blots.

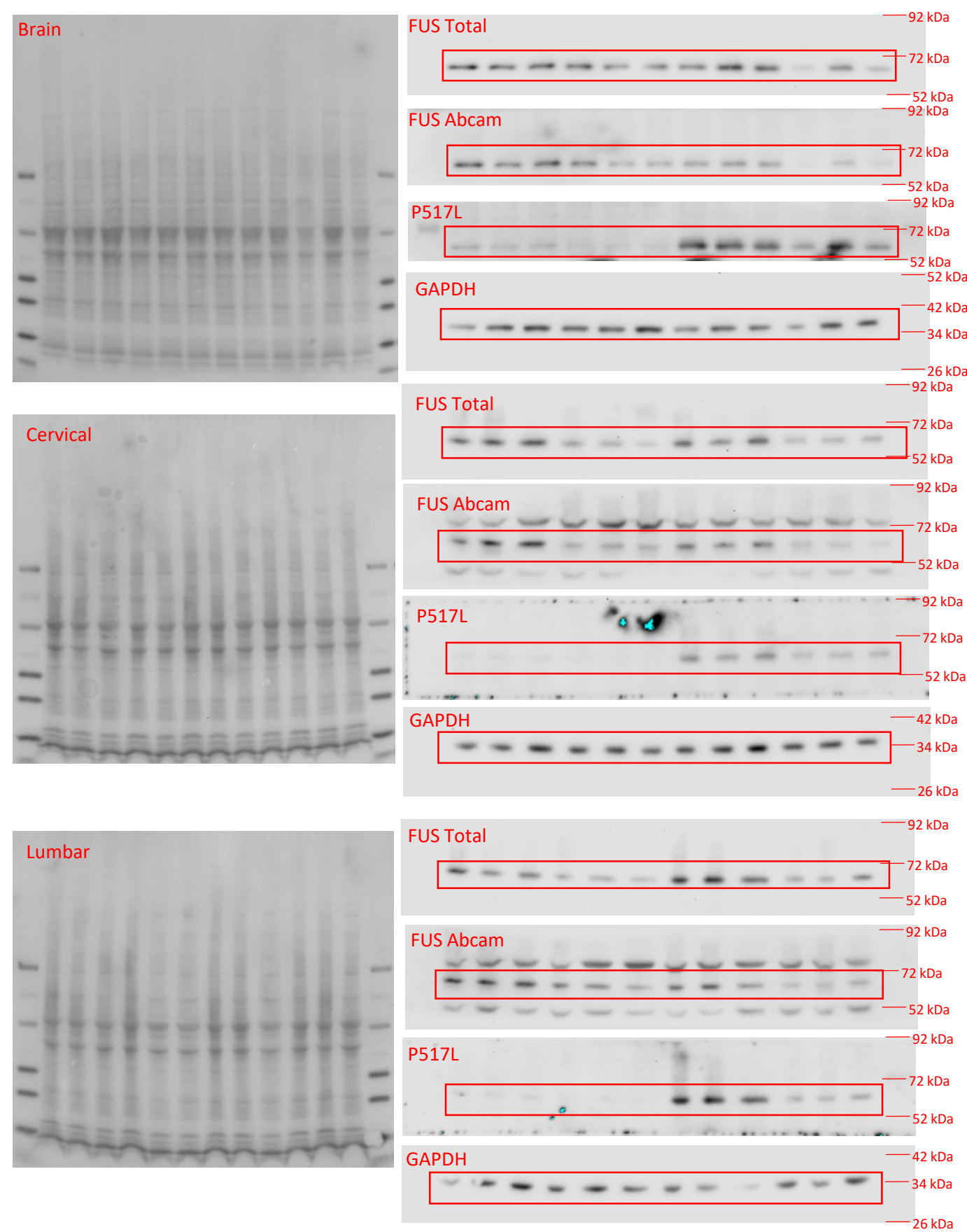

Figure 4c.

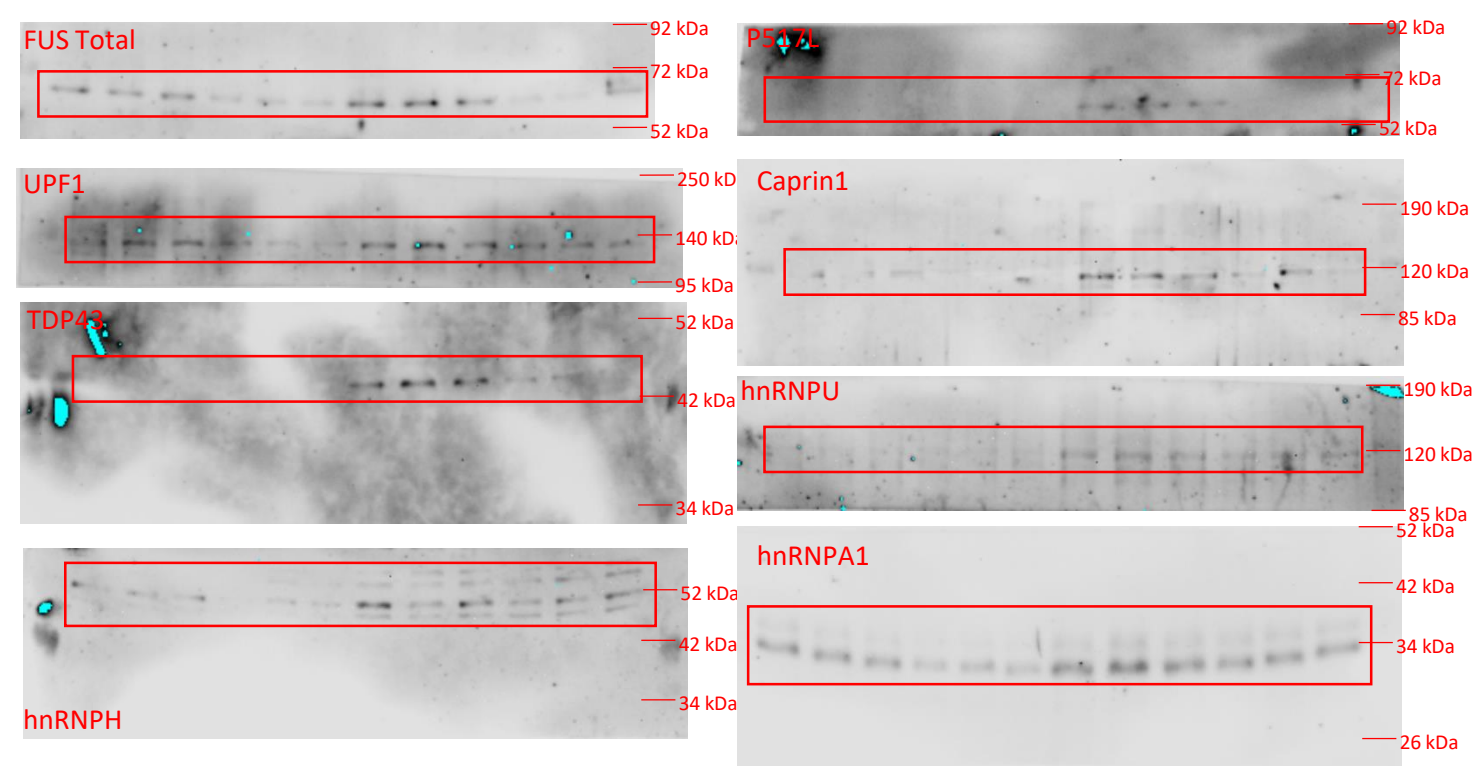

Supplement: Supplementary file 5 — Unprocessed western blots. [file 41591_2021_1615_MOESM5_ESM.pdf]

Figure 5c.

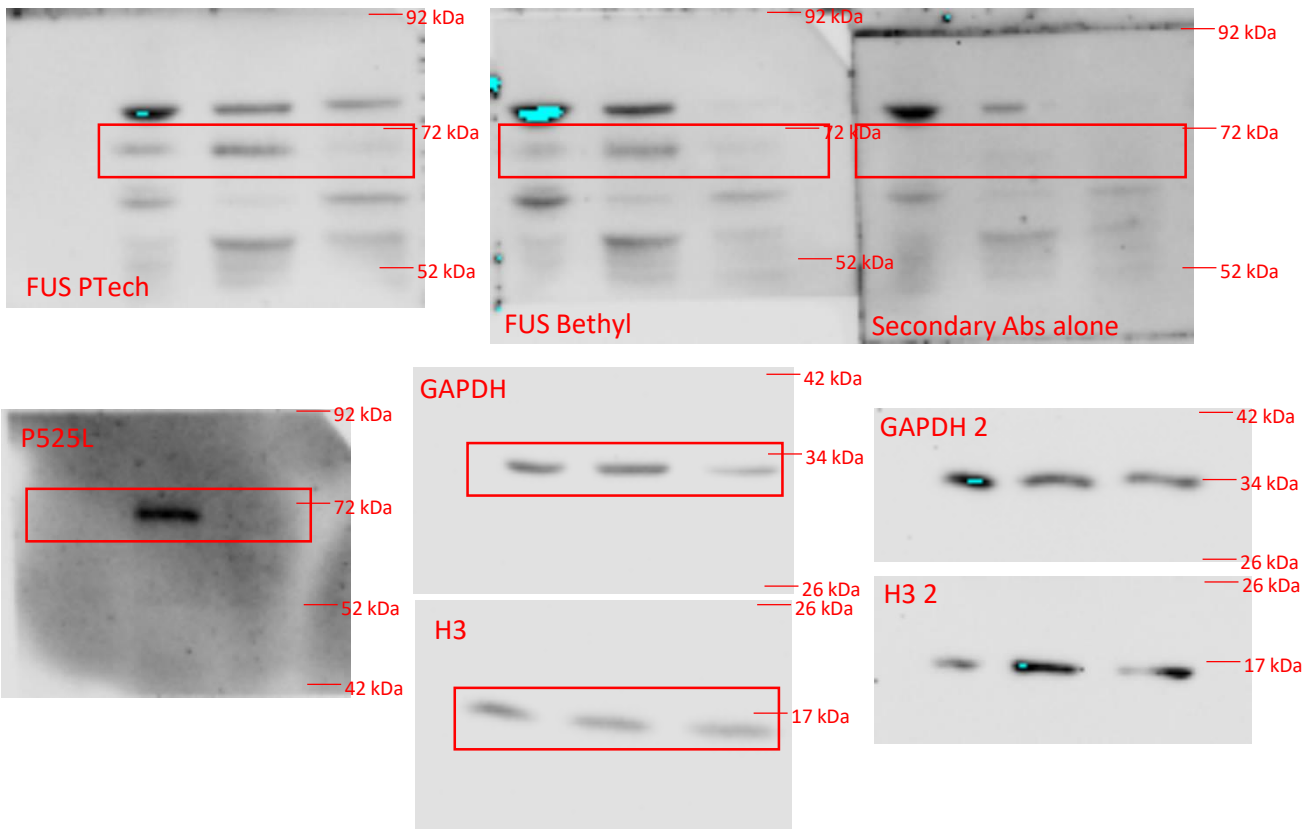

Figure 5d.

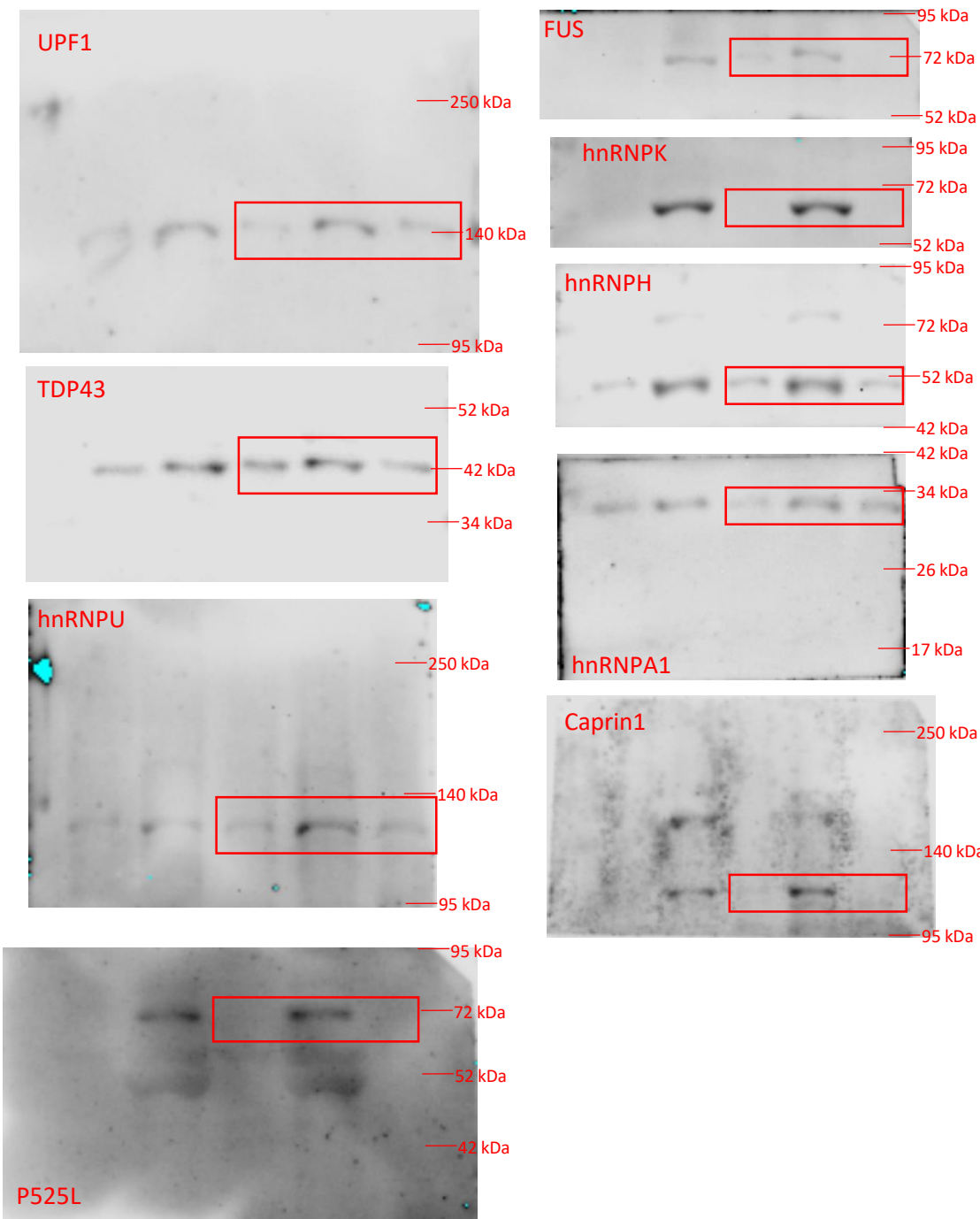

Supplement: Supplementary file 6 — Unprocessed western blots. [file 41591_2021_1615_MOESM6_ESM.pdf]

Extended Data Figure 4a.

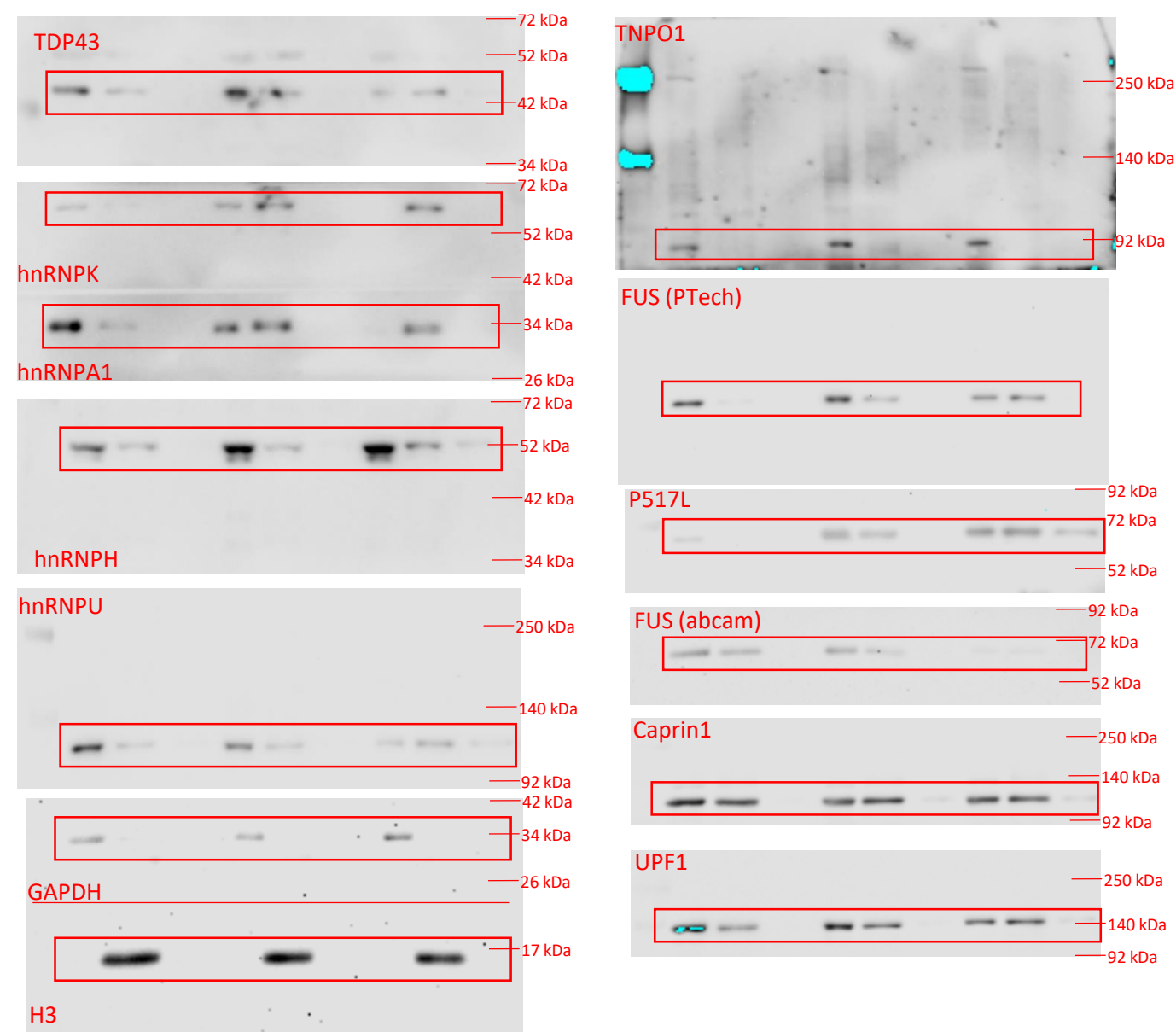

Supplement: Supplementary file 9 — Unprocessed western blots. [file 41591_2021_1615_MOESM9_ESM.pdf]

Uncropped images of Western blots.

Extended Data Figure 6c.

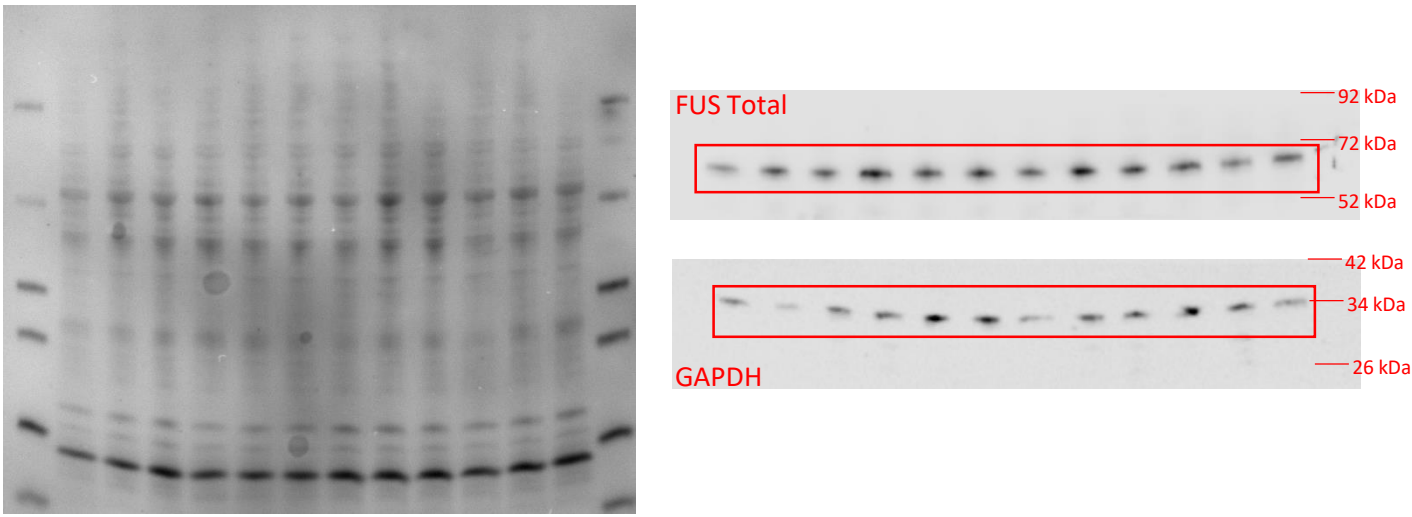

Supplement: Supplementary file 10 — Unprocessed western blots. [file 41591_2021_1615_MOESM10_ESM.pdf]
