# Supplementary material for: Antisense oligonucleotide silencing of FUS expression as a therapeutic approach in amyotrophic lateral sclerosis
Source: Nat Med. 2022 Jan 24;28(1):104–16. doi: 10.1038/s41591-021-01615-z (PMC8799464; doi:10.1038/s41591-021-01615-z)
Supplement: Supplementary file 7 — Unprocessed western blots. [file 41591_2021_1615_MOESM7_ESM.pdf]

Uncropped images of Western blots.

Extended Data Figure 1c.

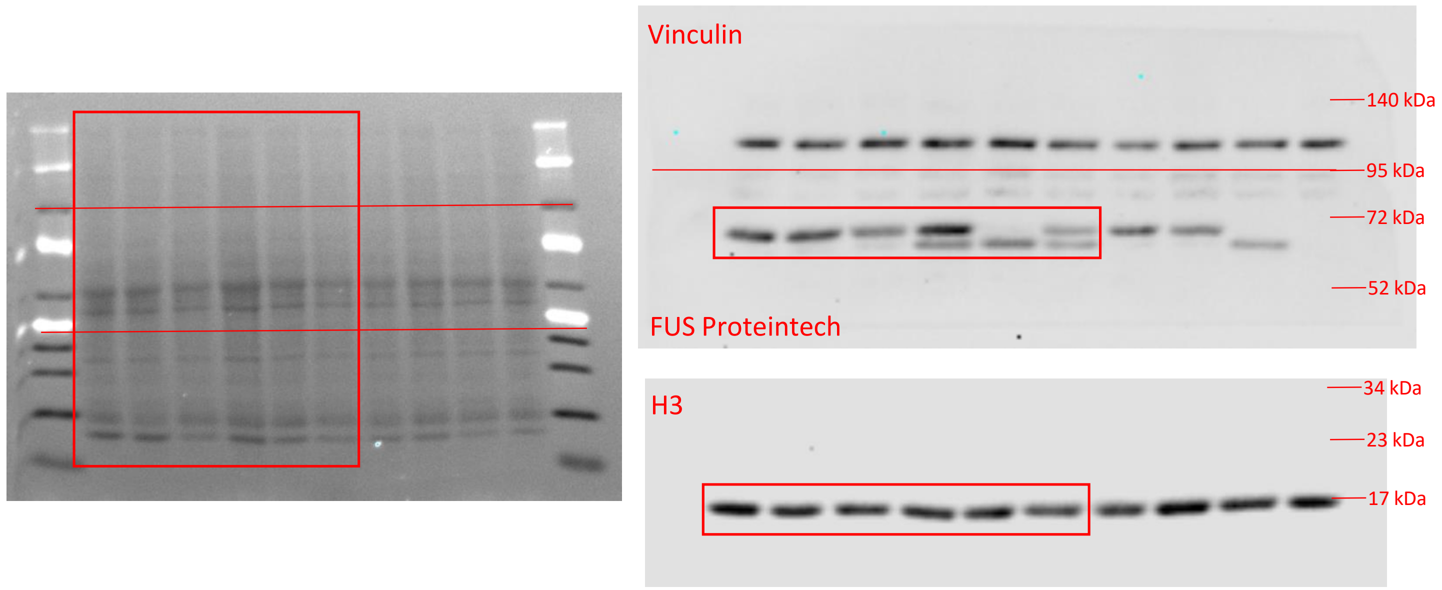

Ponceau stain and uncropped WB images used for Extended Data Figure 1c. The order of the samples is WT/WT, P517L/WT, P517L/P517L, Δ14/WT, Δ14/Δ14, P517L/Δ14, WT/KO, P517L/KO, Δ14/KO, and KO/KO. The last 4 samples were not included. The membrane was cut across into several strips at 95kD and below 42 kDa and probed with Vinculin, FUS (top panel) and H3 (lower panel) antibodies.
