# Supplementary material for: Antisense oligonucleotide silencing of FUS expression as a therapeutic approach in amyotrophic lateral sclerosis
Source: Nat Med. 2022 Jan 24;28(1):104–16. doi: 10.1038/s41591-021-01615-z (PMC8799464; doi:10.1038/s41591-021-01615-z)
Supplement: Supplementary file 8 — Unprocessed western blots. [file 41591_2021_1615_MOESM8_ESM.pdf]

Uncropped images of Western blots.

Extended Data Figure 2g.

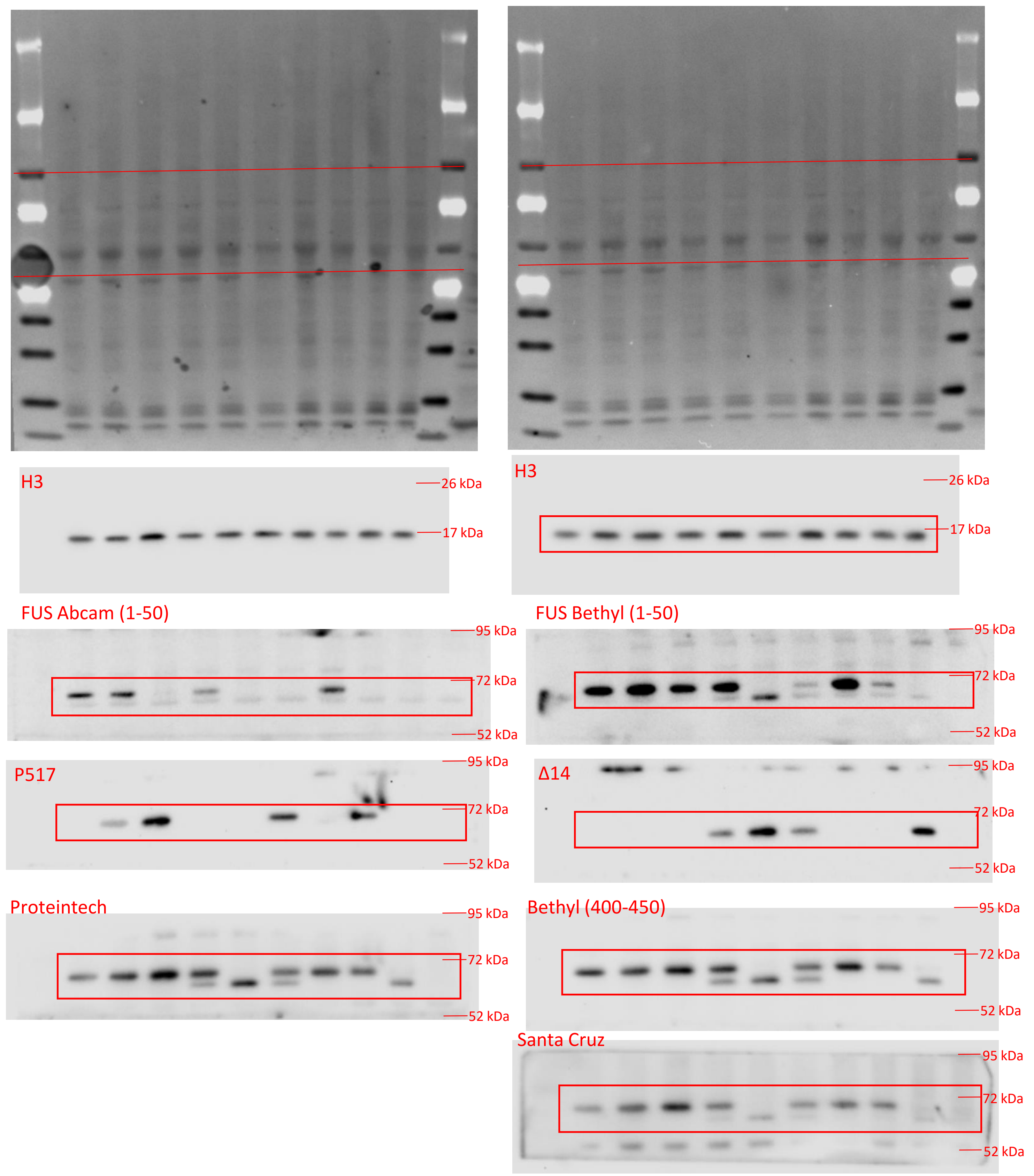

Ponceau stain and uncropped WB images used for Extended Data Figure 2g. Two membranes with identical sample loading were used. The order of the samples is WT/WT, P517L/WT, P517L/P517L, Δ14/WT, Δ14/Δ14, P517L/Δ14, WT/KO, P517L/KO, Δ14/KO, and KO/KO. The remaining lanes after the last ladder contained unrelated samples. The membranes were cut across into several strips at 95kD and above 42 kDa and probed with H3 and different FUS antibodies. The non-specific band in FUS abcam was adjusted by changing intensity levels across the entire image based on the KO/KO condition.
