# Supplementary material for: Antisense oligonucleotide silencing of FUS expression as a therapeutic approach in amyotrophic lateral sclerosis
Source: Nat Med. 2022 Jan 24;28(1):104–16. doi: 10.1038/s41591-021-01615-z (PMC8799464; doi:10.1038/s41591-021-01615-z)
Supplement: Supplementary file 11 — Unprocessed western blots. [file 41591_2021_1615_MOESM11_ESM.pdf]

Uncropped images of Western blots.

Extended Data Figure 7b.

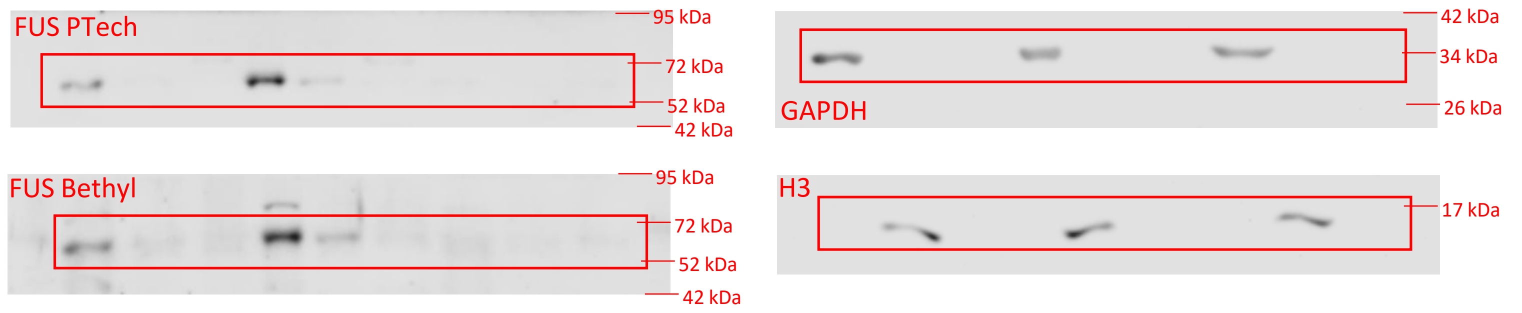

Extended Data Figure 7c.

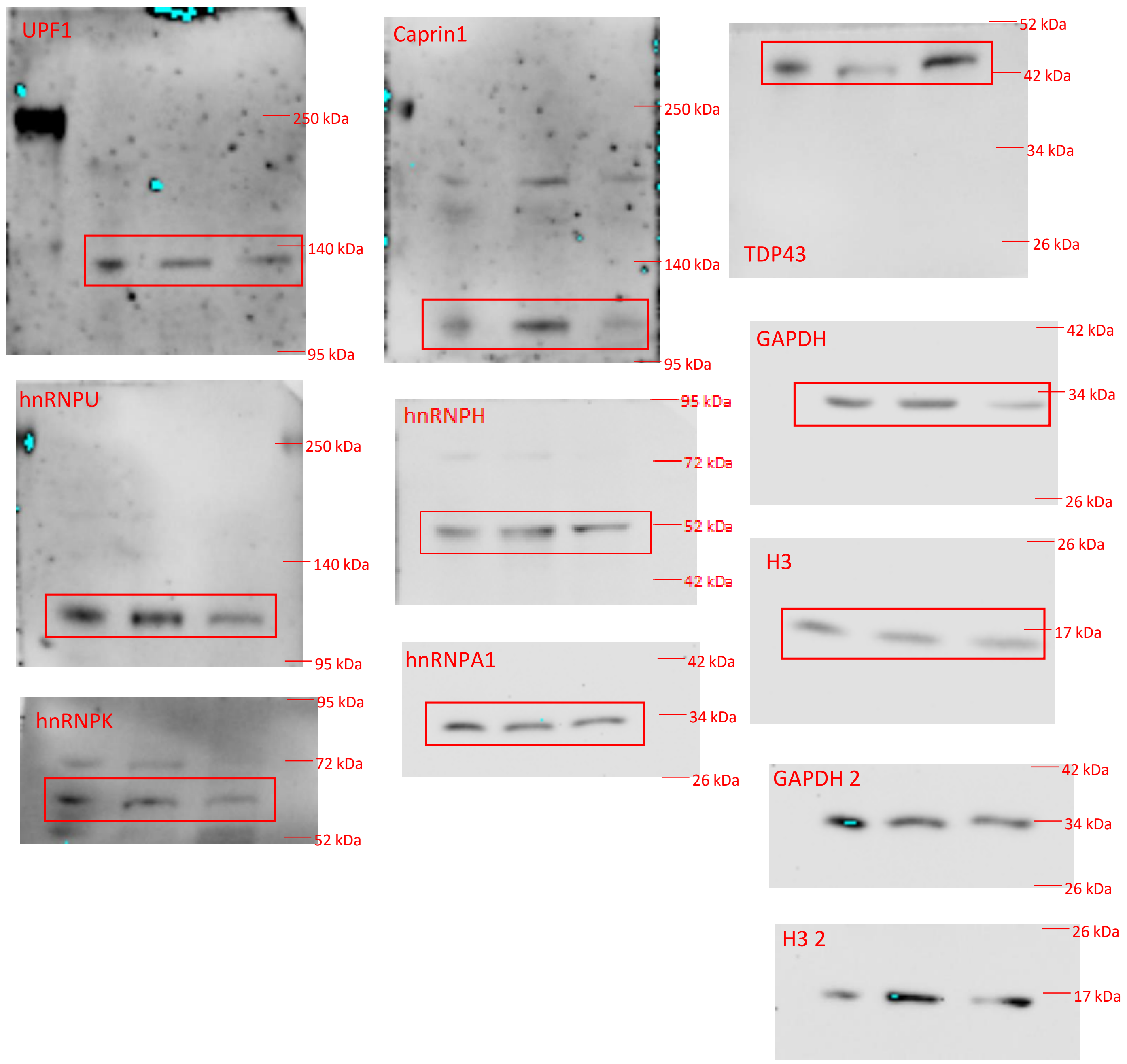

GAPDH and H3 blots also appear in Figure 5c, they are the loading controls for both Figure 5c and Extended Data Figure 7c.
